# The use of mid-regional proadrenomedullin to identify disease severity and treatment response to sepsis - a secondary analysis of a large randomised controlled trial

**DOI:** 10.1186/s13054-018-2001-5

**Published:** 2018-03-21

**Authors:** Gunnar Elke, Frank Bloos, Darius Cameron Wilson, Frank Martin Brunkhorst, Josef Briegel, Konrad Reinhart, Markus Loeffler, Stefan Kluge, Axel Nierhaus, Ulrich Jaschinski, Onnen Moerer, Andreas Weyland, Patrick Meybohm, the SepNet Critical Care Trials Group

**Affiliations:** 1 Department of Anaesthesiology and Intensive Care Medicine, University Medical Center Schleswig-Holstein, Campus Kiel, Arnold-Heller-Str. 3 Haus 12, 24105 Kiel, Germany; 2 Department of Anesthesiology and Intensive Care Medicine, Jena University Hospital, Am Klinikum 1, 07747 Jena, Germany; 3 Center for Sepsis Control & Care (CSCC), Jena University Hospital, Am Klinikum 1, 07747 Jena, Germany; 4 B·R·A·H·M·S GmbH, Hennigsdorf, Neuendorfstr. 25, 16761 Hennigsdorf, Germany; 5 Department of Anaesthesiology, University Hospital Munich, Marchioninistrasse 15, 81377 Munich, Germany; 6 Clinical Trial Centre Leipzig, University of Leipzig, Härtelstraße 16-18, 04107 Leipzig, Germany; 7 Department of Intensive Care Medicine, University Hospital Hamburg-Eppendorf, Martinistr. 52, 20246 Hamburg, Germany; 8 Department of Anaesthesiology and Surgical Intensive Care Medicine, Hospital Augsburg, Stenglinstrasse 2, 86156 Augsburg, Germany; 9 Department of Anaesthesiology, University Hospital Göttingen, Robert-Koch-Str. 40, 37099 Göttingen, Germany; 10 University Department for Anesthesia, Intensive and Emergency Medicine and Pain Management, Hospital Oldenburg, Rahel-Straus-Str. 10, 26133 Oldenburg, Germany; 11 Department of Anaesthesiology, Intensive Care Medicine and Pain Therapy, University Hospital Frankfurt, Theodor-Stern-Kai 7, 60590 Frankfurt am Main, Germany

**Keywords:** MR-proADM, Biomarkers, Sepsis, Mortality, SOFA, Septic shock

## Abstract

**Background:**

This study assessed the ability of mid-regional proadrenomedullin (MR-proADM) in comparison to conventional biomarkers (procalcitonin (PCT), lactate, C-reactive protein) and clinical scores to identify disease severity in patients with sepsis.

**Methods:**

This is a secondary analysis of a randomised controlled trial in patients with severe sepsis or septic shock across 33 German intensive care units. The association between biomarkers and clinical scores with mortality was assessed by Cox regression analysis, area under the receiver operating characteristic and Kaplan-Meier curves. Patients were stratified into three severity groups (low, intermediate, high) for all biomarkers and scores based on cutoffs with either a 90% sensitivity or specificity.

**Results:**

1089 patients with a 28-day mortality rate of 26.9% were analysed. According to the Sepsis-3 definition, 41.2% and 58.8% fulfilled the criteria for sepsis and septic shock, with respective mortality rates of 20.0% and 32.1%. MR-proADM had the strongest association with mortality across all Sepsis-1 and Sepsis-3 subgroups and could facilitate a more accurate classification of low (e.g. MR-proADM vs. SOFA: *N* = 265 vs. 232; 9.8% vs. 13.8% mortality) and high (e.g. MR-proADM vs. SOFA: *N* = 161 vs. 155; 55.9% vs. 41.3% mortality) disease severity. Patients with decreasing PCT concentrations of either ≥ 20% (baseline to day 1) or ≥ 50% (baseline to day 4) but continuously high MR-proADM concentrations had a significantly increased mortality risk (HR (95% CI): 19.1 (8.0–45.9) and 43.1 (10.1–184.0)).

**Conclusions:**

MR-proADM identifies disease severity and treatment response more accurately than established biomarkers and scores, adding additional information to facilitate rapid clinical decision-making and improve personalised sepsis treatment.

**Electronic supplementary material:**

The online version of this article (10.1186/s13054-018-2001-5) contains supplementary material, which is available to authorized users.

## Background

The incidence of sepsis has continued to escalate rapidly in hospitalized patients [1], with mortality rates of between 10% and 54%, depending on disease severity [2, 3]. A prompt assessment of the infectious load and disease severity in the early stages of sepsis is therefore crucial in order to provide a rapid diagnostic and therapeutic response [4]. In addition, an accurate assessment of disease severity may help to guide physicians in making efficient intensive care unit (ICU) discharge decisions. However, to date, no diagnostic markers allow for a reliable severity assessment to be made [4]. Clinical scores such as the Sequential Organ Failure Assessment (SOFA) score, Acute Physiological and Chronic Health Evaluation (APACHE) II, and the Simplified Acute Physiological Score (SAPS) II have previously been developed for this purpose [5]. However, such scores may not promptly capture individual organ system dysfunction [4], and incorporation into daily routine is hampered by their relative complexity. The use of biomarkers might therefore satisfy this unmet clinical need.

Mid-regional proadrenomedullin (MR-proADM) is a peptide generated by multiple tissues in order to stabilise the microcirculation and protect against endothelial permeability [6–11], both of which are widely acknowledged to play a significant role in the pathophysiological host response to sepsis [12, 13]. Indeed, MR-proADM levels are rapidly induced during the initial stages of sepsis development following burns [14] and neurological disorders [15], in response to invasive fungal infections in patients with septic shock [16], and in other conditions such as lower respiratory tract infections [17–19], lung transplantation [20] and thoracic surgery [21]. Thus, MR-proADM may be of significant clinical utility in the early risk stratification of patients with sepsis. However, supporting data from large patient populations are rare.

In this secondary analysis of a previous randomised controlled trial [22], we aimed to investigate MR-proADM performance in comparison to a range of biomarkers (procalcitonin, lactate, C-reactive protein) and clinical scores (SOFA, APACHE II and SAPS II) in order to (i) make an accurate assessment of disease severity at diagnosis and throughout ICU therapy, (ii) aid in the early assessment of treatment response, and (iii) identify low-risk patients eligible for an early ICU discharge to a step-down unit.

## Methods

### Study design and patients

This is a secondary analysis of the randomised *Placebo-Controlled Trial of Sodium Selenite and Procalcitonin Guided Antimicrobial Therapy in Severe Sepsis (SISPCT)* trial, performed across 33 German multidisciplinary ICUs from November 2009 until February 2013 [22]. Inclusion criteria were adults ≥ 18 years of age presenting with new-onset severe sepsis or septic shock (≤ 24 h), according to the Sepsis-1 definition [23]. The study protocol was approved by the ethics board of Jena University Hospital. Written informed consent was obtained from all patients or their legal representatives. For the purpose of this analysis, patients were further classified according to the Sepsis-3 definitions [4]. Details of the SISPCT study design, data collection and management were described previously [22].

### Biomarker measurements

Patients were enrolled up to 24 h after diagnosis of severe sepsis or septic shock, and serum C-reactive protein (CRP) and lactate concentrations measured immediately thereafter. Additional blood samples were collected at baseline and on days 1, 4, 7 and 10 and stored at the central study laboratory in Jena, Germany, at - 80 °C. MR-proADM and procalcitonin (PCT) plasma concentrations were measured retrospectively (Kryptor®, Thermo Fisher Scientific, Germany) with a limit of detection of 0.05 nmol/L and 0.02 ng/ml, respectively. APACHE II and SAPS II scores were calculated at baseline, whilst SOFA scores were calculated at all time points.

### Statistical analysis

The onset of either severe sepsis or septic shock was considered as day 0 (baseline), irrespective of the prior duration of hospital or ICU length of stay (LOS). Differences in demographic and clinical characteristics with regards to 28-day mortality were assessed at baseline using the chi-square (χ^2^) test for categorical variables, and Student’s *t* test or the Mann-Whitney U test for continuous variables, depending on distribution normality. Normally and non-normally distributed variables were expressed as mean (standard deviation) and median (first quartile to third quartile), respectively. The association between mortality and each biomarker and clinical score was assessed using area under the receiver operating characteristic curves (AUROC) and Cox regression analysis, with multivariate analysis corrected for age, the presence of comorbidities and septic shock. Net reclassification improvement (NRI) was used to evaluate the additional performance of MR-proADM to individual markers or scores across the total population and surviving and non-surviving patient groups [24]. For each biomarker and clinical score at each time point, two cutoffs with a predefined sensitivity or specificity close to 90% were derived from the AUROCs, allowing patients to be classified into three severity subgroups (low, intermediate and high). A subgroup of clinically stable patients was subsequently identified with an absence of any ICU-associated procedures or complications, which included focus eradication procedures, emergency surgery, new infections, transfusion of blood products, infusion of colloids, invasive mechanical ventilation, renal/liver replacement or vasopressor therapy, and a deterioration in the patient’s general clinical signs. Patients within this group with low MR-proADM concentrations (which had not increased since the previous measurement) were further analysed. Mortality rates and average LOS were calculated in both groups and compared to the patient group discharged at each specific time point.

Finally, the response to ICU treatment was investigated by constructing models of PCT, MR-proADM and SOFA kinetics over time. Multivariate logistic regression was used to assess the relationship of biomarkers and their interactions at baseline, day 1 and day 4 with mortality, considering both absolute concentrations and the delta change between time points. Accordingly, two models stratifying patients based on PCT decreases of ≥20% or <20% from baseline to day 1 and ≥50% or <50% from baseline to day 4 (based on a previous model [22]), and three models stratifying patients with either decreasing (≥ 2 points), stable (< 2 point change) or increasing (≥ 2 points) SOFA scores from baseline to day 1, were constructed. Patient subgroups were subsequently identified according to MR-proADM concentrations, and respective mortality rates calculated. The risk of mortality within each subgroup in comparison to other subgroups was calculated by Cox regression analysis and illustrated by Kaplan-Meier curves. The predicted risk of developing new infections and the requirement for focus control procedures and emergency surgery over days 4 to 7 was subsequently investigated. All data were analysed using the statistics software R (version 3.1.2).

## Results

A total of 1089 patients with either severe sepsis (number (*N*) = 142; 13.0%) or septic shock (*N* = 947; 87.0%) were analysed. The 28-day all-cause mortality rate was 26.9% with a hospital mortality rate of 33.4%. Of these patients, 439 (41.2%) and 627 (58.8%) fulfilled the criteria for sepsis and septic shock according to the Sepsis-3 definition, with 28-day and hospital mortality rates of 20.0% and 24.4% (sepsis) and 32.1% and 40.4% (septic shock), respectively. Patient characteristics upon study enrollment for 28-day mortality are summarized in Table 1. The most common causes of mortality included sepsis-induced multiple organ failure (*N* = 132; 45.7%), refractory septic shock (*N* = 54; 18.7%), death due to pre-existing illness (*N* = 35; 12.1%) and acute respiratory insufficiency (*N* = 17; 5.9%). Other causes not directly related to sepsis accounted for a mortality rate of 8.6%. A limitation of therapy was applied in 3.4% of patients. Supplementary results on infectious foci and microbial identification are reported in Additional file 1. In general, non-surviving patients had significantly higher concentrations of MR-proADM, PCT and lactate, as well as higher SOFA, APACHE II and SAPS II scores than survivors. CRP concentrations were not significantly different.

**Table 1:** Clinical patient characteristics at baseline with regards to survival up to 28 days

	Total (*N* = 1076)	Survivors (*N* = 787)	Non-Survivors (*N* = 289)	*p* value
Age (years) (mean, SD)	65.7 (13.7)	64.3 (14.0)	69.5 (12.0)	<0.001
Male gender (*N*, %)	681 (63.3%)	510 (64.8%)	171 (59.2%)	0.091
Definitions of sepsis and length of stay				
Sepsis-1, severe sepsis (*N*, %)	139 (12.9%)	109 (13.9%)	30 (10.4%)	0.125
Sepsis-1, septic shock (*N*, %)	937 (87.1%)	678 (86.2%)	259 (89.6%)	0.125
Sepsis-3, sepsis (*N*, %)	439 (41.2%)	351 (45.2%)	88 (30.4%)	<0.001
Sepsis-3, septic shock (*N*, %)	627 (58.8%)	426 (54.8%)	201 (69.6%)	<0.001
ICU length of stay (days) (median, IQR)	12 (6 - 23)	13 (7 - 26)	8 (4 - 15)	<0.001
Hospital length of stay (days) (median, IQR)	28 (17 - 45)	34 (22 - 51)	14 (7 - 23)	<0.001
Pre-existing comorbidities				
History of diabetes mellitus (*N*, %)	280 (26.0%)	188 (23.9%)	92 (31.8%)	0.009
Heart failure (*N*, %)	230 (21.4%)	150 (19.1%)	80 (27.7%)	0.003
Renal dysfunction (*N*, %)	217 (20.2%)	135 (17.2%)	82 (28.4%)	<0.001
COPD (*N*, %)	131 (12.2%)	90 (11.4%)	41 (14.2%)	0.228
Liver cirrhosis (*N*, %)	50 (4.7%)	27 (3.4%)	23 (8.0%)	0.003
History of cancer (*N*, %)	319 (29.7%)	224 (28.5%)	95 (32.9%)	0.163
Immunosuppression (*N*, %)	46 (4.3%)	30 (3.8%)	16 (5.5%)	0.227
Microbiology				
Gram positive (*N*, %)	146 (13.6%)	113 (14.4%)	33 (11.4%)	0.205
Gram negative (*N*, %)	132 (12.3%)	95 (12.1%)	37 (12.8%)	0.747
Fungal (*N*, %)	51 (4.7%)	37 (4.7%)	14 (4.8%)	0.922
Gram positive and negative (*N*, %)	183 (17.0%)	133 (16.9%)	50 (17.3%)	0.877
Gram positive and fungal (*N*, %)	92 (8.6%)	68 (8.6%)	24 (8.3%)	0.861
Gram negative and fungal (*N*, %)	51 (4.7%)	35 (4.5%)	16 (5.5%)	0.463
Gram positive and negative and fungal (*N*, %)	115 (10.7%)	81 (10.3%)	34 (11.8%)	0.492
Origin of infection				
Pneumonia (*N*, %)	453 (43.7%)	327 (42.9%)	126 (46.0%)	0.380
Upper or lower respiratory (*N*, %)	44 (4.3%)	29 (3.8%)	15 (5.5%)	0.252
Thoracic (*N*, %)	44 (4.3%)	35 (4.6%)	9 (3.3%)	0.344
Bones/soft tissue (*N*, %)	78 (7.5%)	56 (7.4%)	22 (8.0%)	0.716
Gastrointestinal (*N*, %)	80 (7.7%)	68 (8.9%)	12 (4.4%)	0.011
Catheter associated (*N*, %)	30 (2.9%)	18 (2.4%)	12 (4.4%)	0.102
Surgical wound (*N*, %)	41 (4.0%)	31 (4.1%)	10 (3.7%)	0.759
Intraabdominal (*N*, %)	375 (36.2%)	276 (36.2%)	99 (36.1%)	0.979
Cardiovascular (*N*, %)	6 (0.6%)	4 (0.5%)	2 (0.7%)	0.708
Urogenital (*N*, %)	99 (9.6%)	70 (9.2%)	29 (10.6%)	0.503
Central nervous system (*N*, %)	3 (0.3%)	2 (0.3%)	1 (0.4%)	0.792
Bacteraemia (*N*, %)	31 (3.0%)	20 (2.6%)	11 (4.0%)	0.261
Organ dysfunction				
Neurological (*N*, %)	348 (32.3%)	240 (30.5%)	108 (37.4%)	0.034
Respiratory (*N*, %)	486 (45.2%)	350 (44.5%)	136 (47.1%)	0.450
Cardiovascular (*N*, %)	829 (77.0%)	584 (74.2%)	245 (84.8%)	<0.001
Renal (*N*, %)	382 (35.5%)	249 (31.6%)	133 (46.0%)	<0.001
Haematological (*N*, %)	156 (14.5%)	89 (11.3%)	67 (23.2%)	<0.001
Gastrointestinal (*N*, %)	387 (36.0%)	271 (34.4%)	116 (40.1%)	0.086
Metabolic (*N*, %)	718 (66.7%)	504 (64.0%)	214 (74.1%)	0.002
Other organ dysfunction (*N*, %)	499 (46.4%)	380 (48.3%)	119 (41.2%)	0.038
Treatment upon sepsis diagnosis				
Invasive mechanical ventilation (*N*, %)	789 (73.3%)	567 (72.1%)	222 (76.8%)	0.113
Non-invasive mechanical ventilation (*N*, %)	64 (5.9%)	46 (5.8%)	18 (6.2%)	0.815
Renal replacement therapy (*N*, %)	326 (30.8%)	158 (20.5%)	168 (58.1%)	<0.001
Vasopressor use (*N*, %)	980 (91.1%)	712 (90.5%)	268 (92.7%)	0.239
Biomarker and severity scores				
MR-proADM (nmol/L) (median, IQR)	5.0 (2.6–8.8)	4.0 (2.3–7.2)	8.2 (5.2–12.6)	<0.001
PCT (ng/mL) (median, IQR)	7.4 (1.6–26.9)	6.6 (1.4–25.1)	9.3 (2.6–31.8)	0.033
Lactate (mmol/L) (median, IQR)	2.7 (1.6–4.7)	2.4 (1.5–4.0)	3.7 (2.1–7.2)	<0.001
CRP (mg/L) (median, IQR)	188 (120.9–282)	189 (120.5–277.4)	188 (122–287)	0.773
SOFA (points) (mean, SD)	10.02 (3.33)	9.58 (3.18)	11.22 (3.43)	<0.001
SAPS II (points) (mean, SD)	63.27 (14.18)	61.08 (13.71)	69.24 (13.74)	<0.001
APACHE II (points) (mean, SD)	24.24 (7.60)	23.05 (7.37)	27.49 (7.28)	<0.001

Data are presented as absolute numbers with percentages in brackets, indicating the proportion of surviving and non-surviving patients at 28 days

*APACHE II* Acute Physiological and Chronic Health Evaluation II score, *COPD* chronic obstructive pulmonary disease, *CRP* C-reactive protein, *ICU* intensive care unit, *MR-proADM* mid-regional proadrenomedullin, *N* number, *PCT* procalcitonin, *SAPS* *II* Simplified Acute Physiological Score II, *SOFA* Sequential Organ Failure Assessment score

### Assocation between biomarkers and clinical scores with mortality at baseline

**Fig. 1:**
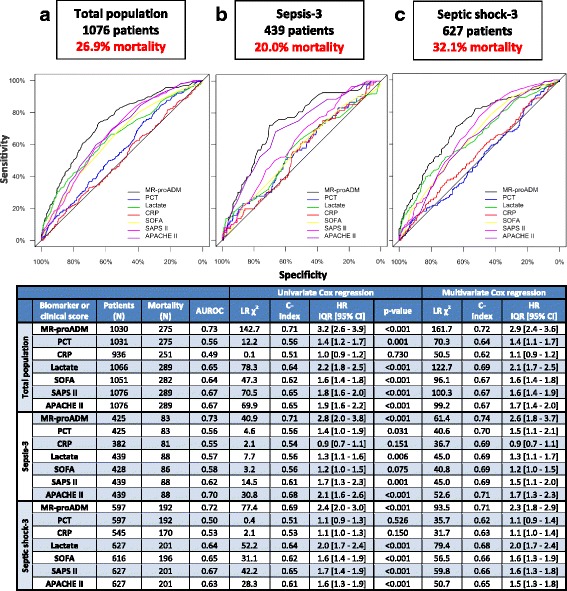
Prediction of 28-day mortality at baseline. Association between biomarkers and clinical scores with mortality at baseline, with respective AUROC and Cox regression analyses across the total patient population (**a**), Sepsis-3 (**b**) and Septic shock-3 (**c**) subgroups. All multivariate analyses for 28-day mortality were significant (*p* < 0.001). *APACHE II* Acute Physiological and Chronic Health Evaluation II score, *AUROC* area under the receiver operating characteristic curve, *CI* confidence interval, *CRP* C-reactive protein, *HR* hazard ratio, *IQR* interquartile range, *MR-proADM* mid-regional proadrenomedullin, *N* number, *PCT* procalcitonin, *SAPS II*, *Simplified Acute Physiological Score II*, *SOFA* Sequential Organ Failure Assessment

AUROC, univariate and multivariate Cox regression analyses indicated that MR-proADM had the strongest association with 28-day mortality across the total patient population, and within the Sepsis-1 (severe sepsis and septic shock hazard ratio (HR) and interquartile range (IQR) (95% confidence interval (CI)): 2.46 (1.45–4.15) and 3.02 (2.48–3.69)) and Sepsis-3 (sepsis and septic shock HR IQR (95% CI): 2.80 (2.04–3.84)) and 2.41 (1.97–2.96); Fig. 1) subgroups. Similar results were found for 7-day, 90-day, ICU and hospital mortality prediction in the total patient population (Table 2). The addition of MR-proADM to all possible biomarker and clinical score combinations (*N* = 63) significantly increased prognostic performance according to likelihood ratio (LR) χ^2^ analysis within the bivariate and multivariate models (Additional file 1: Table S1). There were also significant increases in the AUROCs for individual biomarkers and scores (Additional file 1: Table S2). Finally, net reclassification improvement analysis resulted in a more accurate classification following the addition of MR-proADM to all biomarkers and scores (Additional file 1: Table S3), and to an existing model of PCT and SOFA in the total population (NRI (95% CI): 0.72 (0.58–0.83)), surviving (NRI (95% CI): 0.32 (0.25–0.39)) and non-surviving (NRI (95% CI)): 0.40 (0.29–0.47)) patient subgroups.

**Table 2:** Survival analysis for 7-day, 90-day, ICU and hospital mortality

					Univariate Cox regression				Multivariate Cox regression		
	Biomarker or clinical score	Patients (N)	Mortality (N)	AUROC	LR χ^2^	C-index	HR IQR (95% CI)	*p* value	LR χ^2^	C-index	HR IQR (95% CI)
7-day mortality	MR-proADM	1037	131	0.72	71.6	0.71	3.3 (2.4–4.3)	<0.001	82.1	0.73	3.4 (2.5–4.6)
	PCT	1038	131	0.58	9.7	0.58	1.5 (1.2–2.0)	0.002	28.4	0.64	1.6 (1.2–2.1)
	CRP	943	111	0.55	1.2	0.55	1.1 (0.9–1.4)	0.284	16.6	0.62	1.2 (0.9–1.4)
	Lactate	1074	135	0.72	86.0	0.71	3.1 (2.4–3.9)	<0.001	99.1	0.73	3.1 (2.4–4.0)
	SOFA	1059	130	0.63	25.5	0.63	1.7 (1.4–2.0)	<0.001	41.0	0.67	1.7 (1.4–2.1)
	SAPS II	1085	135	0.66	38.5	0.66	1.8 (1.5–2.2)	<0.001	50.1	0.67	1.8 (1.5–2.2)
	APACHE II	1085	135	0.63	24.4	0.63	1.7 (1.4–2.1)	<0.001	37.8	0.65	1.7 (1.4–2.1)
90-day mortality	MR-proADM	1000	379	0.71	146.2	0.68	2.7 (2.3–3.2)	<0.001	194.1	0.71	2.4 (2.0–2.8)
	PCT	1000	379	0.55	11.8	0.55	1.3 (1.1–1.5)	0.001	113.5	0.65	1.3 (1.1–1.5)
	CRP	909	348	0.51	0.2	0.51	1.0 (0.9–1.2)	0.664	92.3	0.64	1.1 (0.9–1.2)
	Lactate	1037	399	0.64	83.2	0.63	2.0 (1.7–2.3)	<0.001	168.8	0.68	1.9 (1.6–2.2)
	SOFA	1021	388	0.62	48.1	0.61	1.5 (1.4–1.7)	<0.001	143.7	0.67	1.5 (1.3–1.7)
	SAPS II	1045	399	0.66	81.1	0.64	1.7 (1.5–1.9)	<0.001	144.4	0.67	1.5 (1.3–1.7)
	APACHE II	1045	399	0.67	86.4	0.64	1.8 (1.6–2.1)	<0.001	146.8	0.67	1.6 (1.4–1.8)
ICU mortality	MR-proADM	1023	264	0.73	136.4	0.73	4.0 (3.1–5.2)	<0.001	158.3	0.75	3.7 (2.8–4.9)
	PCT	1024	264	0.58	18.0	0.58	1.6 (1.3–2.0)	<0.001	73.0	0.67	1.6 (1.3–2.1)
	CRP	928	237	0.54	2.5	0.54	1.1 (1.0–1.3)	0.111	51.4	0.65	1.2 (1.0–1.4)
	Lactate	1059	277	0.66	75.2	0.66	2.4 (2.0–3.0)	<0.001	115.5	0.71	2.4 (1.9–2.9)
	SOFA	1044	270	0.64	48.6	0.64	1.8 (1.5–2.2)	<0.001	95.2	0.69	1.8 (1.5–2.2)
	SAPS II	1070	277	0.65	58.7	0.65	1.9 (1.6–2.3)	<0.001	91.2	0.68	1.8 (1.5–2.2)
	APACHE II	1070	277	0.66	62.5	0.66	2.1 (1.7–2.6)	<0.001	91.6	0.69	1.9 (1.5–2.3)
Hospital mortality	MR-proADM	980	323	0.73	152.0	0.74	4.0 (3.1–5.2)	<0.001	186.8	0.76	3.6 (2.7–4.6)
	PCT	981	323	0.57	15.0	0.57	1.5 (1.2–1.9)	<0.001	96.2	0.68	1.5 (1.2–1.9)
	CRP	891	299	0.52	0.9	0.52	1.1 (0.9–1.3)	0.348	76.0	0.67	1.1 (1.0–1.3)
	Lactate	1016	342	0.66	77.8	0.66	2.4 (2.0–2.9)	<0.001	146.2	0.72	2.3 (1.9–2.9)
	SOFA	1001	333	0.63	41.3	0.63	1.7 (1.4–2.0)	<0.001	118.9	0.70	1.7 (1.4–2.0)
	SAPS II	1027	342	0.65	59.1	0.65	1.9 (1.6–2.2)	<0.001	115.9	0.69	1.7 (1.4–2.0)
	APACHE II	1027	342	0.67	76.7	0.67	2.2 (1.9–2.7)	<0.001	127.1	0.71	1.9 (1.6–2.4)

All multivariate *p* values <0.001, apart from PCT and CRP for 7-day mortality (0.002 and 0.084, respectively)

*APACHE II* Acute Physiological and Chronic Health Evaluation II score, *CI* confidence interval, *CRP* C-reactive protein, *HR* hazard ratio, *IQR* interquartile range, *MR-proADM* mid-regional proadrenomedullin, *N* number, *PCT* procalcitonin, *SAPS I* *I* Simplified Acute Physiological Score II, *SOFA* Sequential Organ Failure Assessment score, *LR* likelihood ratio

### Identification of high-risk patients at baseline

**Fig. 2:**
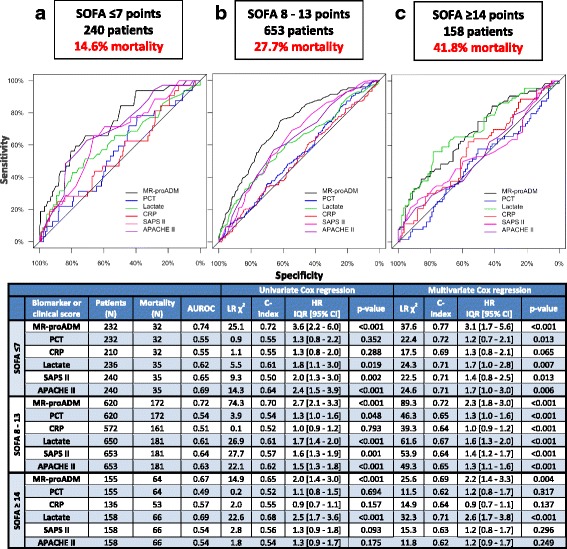
Cox regression and AUROC analysis for 28-day mortality prediction based on SOFA severity levels. Biomarker and clinical score performance in predicting 28-day mortality with respective AUROC and Cox regression analyses in the low (SOFA ≤7) (**a**), moderate (SOFA 8–13) (**b**) and high (SOFA ≥14) (**c**) severity SOFA subgroups. *APACHE II* Acute Physiological and Chronic Health Evaluation II score, *AUROC* area under the receiver operating characteristic curve, *CI* confidence interval, *CRP* C-reactive protein, *HR* hazard ratio, *IQR* interquartile range, *MR-proADM* mid-regional proadrenomedullin, *N* number, *PCT* procalcitonin, *SAPS II* Simplified Acute Physiological Score II, *SOFA* Sequential Organ Failure Assessment

All patients were further stratified into low, intermediate and high SOFA severity levels, and biomarker and clinical score performance in predicting 28-day mortality was assessed in each subgroup. MR-proADM had the highest accuracy among all parameters in the low (SOFA ≤7) and moderate (SOFA 8–13) severity SOFA subgroups (Fig. 2; Additional file 1: Table S4). Two corresponding MR-proADM cutoffs were subsequently calculated to identify low (≤ 2.75 nmol/L) and high (> 10.9 nmol/L) severity patient populations at baseline (Additional file 1: Table S5). Compared to SOFA, a more accurate classification could be made in identifying low (MR-proADM vs. SOFA, *N* = 265 vs. 232; 9.8% vs. 13.8% mortality) and high (MR-proADM vs. SOFA, *N* = 161 vs. 155; 55.9% vs. 41.3% mortality) disease severity patients (Additional file 1: Table S6). A subgroup of 94 patients (9.3%) with high MR-proADM concentrations and corresponding low/intermediate SOFA values had respective 28 and 90-day mortality rates of 57.4% and 68.9%, compared to 19.8% and 30.8% in the remaining low/intermediate SOFA patient population. There were similar patterns for MR-proADM performance in relation to SAPS II, APACHE II and lactate, respectively ( Additional file 1: Supplementary results and Tables S7–S9).

### Identification of low-risk patients throughout ICU stay

MR-proADM had the strongest association with 28-day mortality across all subsequent time points (Additional file 1: Table S10). Across days 4–10, a cutoff of ≤ 2.25 nmol/L identified more patients with a lower mortality rate than the other biomarkers and clinical scores (Additional file 1: Tables S11–S12). Accordingly, 290 low MR-proADM severity patients were identified on day 4, of which 79 (27.2%) were deemed as clinically stable with no increase in MR-proADM concentration from the previous measurement (Table 3). A continuously low MR-proADM concentration from day 1 was identified in 51 (64.6%) patients, whilst a decrease from an intermediate to low severity level was observed in 28 (35.4%) patients. Conversely, patients who maintained MR-proADM concentrations > 2.25 nmol/L at all time points had a significantly higher 28-day mortality risk (Additional file 1: Table S13). The average ICU LOS was 8 (7–10) days, with a 28 and 90-day mortality rate of 0.0% and 1.4%, respectively. In comparison, only 43 patients were actually discharged from the ICU on day 4, with a 28 and 90-day mortality rate of 2.3% and 10.0%, respectively. MR-proADM concentration analysis within this patient group indicated that 52.6%, 42.1% and 5.3% of patients were discharged with low, intermediate and high-severity concentrations, respectively. The results were similar on ICU days 7 and 10.

**Table 3:** Mortality and duration of ICU therapy at different time points

	Patient severity group	Patients (N)	SOFA (points)	Length of stay (days)	28-day mortality (N, %)	90-day mortality (N, %)
Day 4	Total patient population	777	8.4 (4.3)	16 (10–27)	158 (20.3%)	256 (33.9%)
	Clinically stable population	145	4.5 (2.4)	8 (6–11)	10 (6.9%)	22 (15.8%)
	Clinically stable and low MR-proADM population	79	3.6 (1.5)	8 (7–10)	0 (0.0%)	1 (1.4%)
	Actual day-4 discharges	43	3.6 (2.1)	–	1 (2.3%)	4 (10.0%)
Day 7	Total patient population	630	8.0 (4.2)	19 (13–31)	127 (20.2%)	214 (34.9%)
	Clinically stable population	124	3.9 (1.7)	11.5 (9–16)	9 (7.3%)	17 (13.9%)
	Clinically stable and low MR-proADM population	78	3.4 (1.6)	11 (9–14)	1 (1.3%)	4 (5.3%)
	Actual day-7 discharges	36	3.6 (2.6)	–	2 (5.6%)	5 (13.9%)
Day 10	Total patient population	503	7.6 (4.0)	23.5 (17–34.25)	82 (16.3%)	159 (32.6%)
	Clinically stable population	85	3.5 (1.8)	15 (13–22)	9 (10.6%)	14 (17.3%)
	Clinically stable and low MR-proADM population	57	3.2 (1.3)	14 (12.25–19)	1 (1.8%)	2 (3.8%)
	Actual day-10 discharges	29	4.0 (2.6)	–	5 (17.2%)	7 (24.1%)

*N* number, *MR-proADM* mid-regional proadrenomedullin, *SOFA* Sequential Organ Failure Assessment score

### Additional value of MR-proADM in the early identification of treatment response

Multivariate logistic regression for all mortality periods analysed indicated that MR-proADM performance at baseline and day 1 was independent of absolute PCT concentrations or change in PCT between each time point. Results were similar on day 4, with MR-proADM performance independent of delta PCT change. Absolute MR-proADM values had the strongest predictive value for mortality, with delta change in MR-proADM having no significant effect on mortality.

**Fig. 3:**
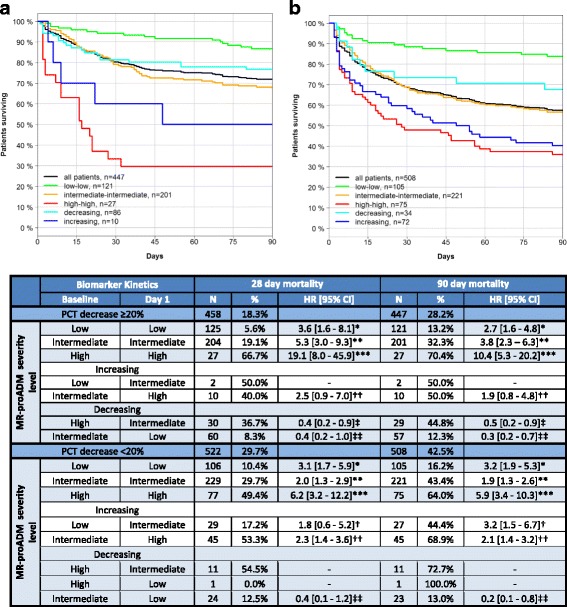
Mortality rates at 28 and 90 days following PCT and MR-proADM kinetics between baseline and day 1. Kaplan-Meier plots illustrate patient subgroups stratified by MR-proADM severity levels for 90-day mortality, based on corresponding PCT concentrations from baseline to day 1, decreasing either by ≥ 20% (**a**) or by < 20% (**b**). Severity levels are grouped as continuously low, intermediate or high, or as a composite for increasing or decreasing MR-proADM levels. Individual hazard ratios for comparisons between patient subgroups are indicated: *continuously intermediate vs. low values; **continuously high vs. intermediate values; ***continuously high vs. low values; †increasing low to intermediate vs. continuously low values; ††increasing intermediate to high vs. continuously intermediate values; ‡decreasing high to intermediate vs. continuously high values; ‡‡decreasing intermediate to low vs. continuously intermediate values. *HR* hazard ratio, *IQR* interquartile range, *MR-proADM* mid-regional proadrenomedullin, *N* number, *PCT* procalcitonin

Accordingly, patients with decreasing PCT concentrations of ≥ 20% from baseline to day 1 (Fig. 3 and Additional file 1: Table S14) or ≥ 50% from baseline to day 4 (Additional file 1: Figure S1 and Table S15) had a 28-day mortality rate of 18.3% (*N* = 458) and 17.1% (*N* = 557), respectively. This decreased to 5.6% (*N* = 125) and 1.8% (*N* = 111) when patients had continuously low levels of MR-proADM, and increased to 66.7% (*N* = 27) and 53.8% (*N* = 39) in the presence of continuously high concentrations (HR (95% CI): 19.1 (8.0–45.9) and 43.1 (10.1–184.0)). A similar model of PCT and SOFA kinetics identified fewer low severity patents who had a higher 28-day mortality rate between baseline and day 1 (*N* = 102; 10.8% mortality) or day 4 (*N* = 64; 4.7% mortality), and identified fewer high severity patients with lower mortality rates between baseline and day 1 (*N* = 16; 50.0% mortality) or day 4 (*N* = 31; 41.9% mortality).

Furthermore, patients with decreasing PCT values of ≥ 50% (baseline to day 4) had a significantly higher risk of developing subsequent nosocomial infections if corresponding MR-proADM concentrations were either continuously high (HR (95% CI): 3.9 (1.5–10.5)) or intermediate (HR (95% CI): 2.4 (1.2–6.8)). In addition, patients with decreasing PCT values of ≥ 50% but increasing intermediate to high MR-proADM concentrations were subsequently more likely to require focus control procedures compared to those with either continuously intermediate (HR (95% CI): 3.2 (1.3–7.6)), intermediate to low (HR (95% CI): 8.7 (3.1–24.8)) or high to intermediate (HR (95% CI): 4.6 (1.4–14.5)) values. When PCT levels failed to decrease by ≥ 50% over the first 4 days of ICU treatment, the risk of requiring emergency surgery was significantly increased if MR-proADM concentrations were either at a continuously high (HR (95% CI): 5.7 (1.5–21.9)) or intermediate (HR (95% CI): 4.2 (1.3–13.2)) level.

Finally, despite undergoing ICU treatment, a stable intermediate SOFA severity level persisted in 260 (26.6%) patients from baseline to day 1, resulting in a 28-day mortality rate of 26.2%. Of these patients, those with continuously low or decreasing MR-proADM concentrations (*N* = 80; 13.8% mortality) had a significantly lower mortality rate compared to those with continuously high or increasing concentrations (*N* = 40; 47.5% mortality; HR (95% CI): 0.1 (0.0–0.4)). Similar MR-proADM subgroups were also identified within the populations with stable low, increasing and decreasing SOFA.

## Discussion

Sepsis remains a major public health concern with high rates of morbidity, mortality and resource use worldwide [25]. Although considerable advances have been made to better define the host response to infection, there is still a lack of specific tools to identify patients at risk of a poor outcome. Accordingly, numerous biomarkers and clinical severity scores have been proposed to fulfil such a requirement, with the SOFA score (representing sepsis-related organ dysfunction) and serum lactate (indicating a deterioration in tissue perfusion) both playing a central role in the recent definition of sepsis [4]. Nevertheless, earlier indicators of developing organ dysfunction or a deteriorating host response are essential in order to guide the most appropriate therapeutic intervention at the earliest opportunity [26, 27].

The novel biomarker, MR-proADM, may fulfil this clinical unmet need, with previous experimental studies showing adrenomedullin to play a significant role in vascular permeability [6], inflammatory mediator and endothelial barrier regulation, and stabilisation of the microcirculation [9, 28, 29] - all of which contribute to the development of organ dysfunction and failure. Accordingly, this secondary analysis of the SISPCT trial [22], for the first time, compared sequential measurements of conventional biomarkers and clinical scores, such as lactate, PCT and SOFA, with those of MR-proADM.

Our results indicate that the initial use of MR-proADM within the first 24 h after sepsis diagnosis resulted in the strongest association with short-term, mid-term and long-term mortality compared to all other biomarkers or clinical scores. Previous studies confirm our findings [30–32], whereas conflicting results [33] may be explained in part by the smaller sample sizes analysed and by other factors highlighted within this study, such as microbial species, origin of infection and previous surgical history, all of which may influence biomarker performance, thus adding to the potential variability of results. Furthermore, our study confirms the results of a previous investigation highlighting the superior performance of MR-proADM in low and intermediate severity organ dysfunction patients with severe sepsis or septic shock [34]. Indeed, Andaluz-Ojeda et al. placed significant importance on patients with low levels of organ dysfunction, since “this group represents either the earliest presentation in the clinical course of sepsis and/or the less severe form of the disease” [34]. The incorporation of MR-proADM into an early sepsis management protocol may therefore help guide early diagnostic interventions and facilitate more intensive treatment in these patient groups before development of any further organ dysfunction. In addition, a reasonable performance across all organ dysfunction, Sepsis-1 and Sepsis-3 subgroups with respect to disease severity further strengthens its clinical utility irrespective of changing definitions or population heterogeneity.

Further analysis of biomarker measurements throughout ICU stay allowed for the effects of therapy to be visualised, resulting in the discrimination of specific patient groups according to host response. Based on the results of this study, two further clinically important uses for MR-proADM can be proposed: (i) the early escalation of therapy in patients at risk of treatment failure and (ii) the de-escalation of treatment and early discharge of low-risk patients.

First, our results revealed a significant benefit in the addition of MR-proADM measurements in the early identification of non-responding patients in order to initiate alternative targeted treatment strategies. Whilst decreasing PCT concentrations are known to indicate the initiation of successful antimicrobial therapy [35, 36], and adherence to a PCT-guided algorithm has been shown to facilitate a reduction in antibiotic use [22, 37, 38], our results revealed a significant benefit in the addition of MR-proADM measurements. The presence of continuously elevated or increasing MR-proADM concentrations in relation to the high sensitivity cutoffs (2.75 and 10.9 nmol/L) identified within this study - despite decreasing PCT concentrations - may provide a prompt indication as to a likely subsequent failure in treatment, and a poor overall outcome. Similar results have been previously found in critically ill febrile patients with cancer, where MR-proADM concentrations were uniquely increased in patients who did not respond to therapy or antibiotic treatment [39]. Such a biomarker constellation may therefore be useful at an early stage of ICU therapy in order to facilitate the earlier initiation of specific interventions, such as focus control and surgical procedures [16], or may potentially aid in the streamlining of antimicrobial agents in patients with sepsis or septic shock [40].

Second, the identification of a population with low disease severity who may be eligible for an early discharge to a step-down setting may be of additional clinical and economic interest [41]. A prompt discharge of patients no longer at risk is essential in maintaining an efficient bed-management workflow as well as well as being of a likely clinical benefit [42]. Our results suggest that the identification of low levels of microcirculatory or vascular damage, as indicated by low MR-proADM concentrations, identifies patients with a very low risk of death in whom early ICU discharge might be possible, and may potentially prevent unnecessary additional diagnostic or interventional procedures [39, 43, 44]. Indeed, similarities have been shown in an earlier randomised controlled trial of 313 patients with suspected lower respiratory tract infections [19]. A non-significant decrease in hospitalization of 0.5 days was identified at the 30-day follow up, although overruling of the MR-proADM algorithm in 34.5% of cases after medical stabilisation resulted in delayed discharge, primarily due to organisational criteria such as further consultant examinations, imaging studies or laboratory results. Such factors should therefore be considered when designing further interventional studies to confirm the results of this analysis.

Interestingly, the discharge of patients with varying MR-proADM concentrations within our study potentially indicated either an incomplete or insufficient treatment, which was consequently reflected in an increased 28 and 90-day mortality rate. Whilst it is unknown whether further ICU treatment for non-microcirculatory, non-life-threatening issues was required, or if beds in a step down-unit were available, such a biomarker-driven approach to ICU discharge in addition to clinician judgement may reduce ICU LOS and improve patient disposition stratification, with accompanied clinical benefits and potential cost savings.

Our analysis has strengths and weaknesses. Biomarker measurements were not collected on a daily basis and were not available earlier than day 1. Given the secondary analysis design of the study, our results should be viewed as exploratory and hypothesis-generating. Admittedly, some of the subgroups analyses involved small patient numbers, revealing the need for future studies to confirm the hypotheses generated. Strengths include the thorough examination of several different subgroups with varying disease severity from a randomised trial database with a high internal validity, and the largest sample size of sepsis patients with MR-proADM measurements to date.

## Conclusions

MR-proADM provides a more accurate disease severity and mortality risk stratification compared to clinically established biomarkers and scores, both on initial diagnosis and over the course of treatment. Changes in MR-proADM kinetics, despite ongoing antimicrobial treatment, may be used to identify patients at risk of treatment failure who may require alternative diagnostic and therapeutic interventions, as well as low severity patients eligible for an early ICU discharge in conjunction with an absence of ICU-specific therapies. Interventional studies to confirm these hypotheses are essential and should be viewed as mandatory before incorporation into routine clinical use.

## Additional file

Additional file 1: Supplementary results. (PDF 5376 kb)

## Abbreviations

APACHE II: Acute Physiological and Chronic Health Evaluation II
AUROC: Area under the receiver operating characteristic curve
CI: Confidence interval
CRP: C-reactive protein
HR: Hazard ratio
ICU: Intensive care unit
IQR: Interquartile range
LOS: Length of stay
LR: Likelihood ratio
MR-proADM: Mid-regional proadrenomedullin
N: Number
NRI: Net reclassification improvement
PCT: Procalcitonin
SAPS II: Simplified Acute Physiological Score II
SISPCT: Placebo-Controlled Trial of Sodium Selenite and Procalcitonin Guided Antimicrobial Therapy in Severe Sepsis
SOFA: Sequential Organ Failure Assessment

## References

[1] Martin GS, Mannino DM, Eaton S, Moss M (2003). The epidemiology of sepsis in the United States from 1979 through 2000. N Engl J Med.

[2] Kaukonen KM, Bailey M, Suzuki S, Pilcher D, Bellomo R (2014). Mortality related to severe sepsis and septic shock among critically ill patients in Australia and New Zealand, 2000-2012. JAMA.

[3] Vincent JL, Sakr Y, Sprung CL (2006). Sepsis in European intensive care units: results of the SOAP study. Crit Care Med.

[4] Singer M, Deutschman CS, Seymour CW (2016). The Third International Consensus Definitions for Sepsis and Septic Shock (Sepsis-3). JAMA.

[5] Vincent JL, Moreno R (2010). Clinical review: scoring systems in the critically ill. Crit Care.

[6] Temmesfeld-Wollbruck B, Brell B, David I (2007). Adrenomedullin reduces vascular hyperpermeability and improves survival in rat septic shock. Intensive Care Med.

[7] Muller-Redetzky HC, Will D, Hellwig K (2014). Mechanical ventilation drives pneumococcal pneumonia into lung injury and sepsis in mice: protection by adrenomedullin. Crit Care.

[8] Carrizo GJ, Wu R, Cui X, Dwivedi AJ, Simms HH, Wang P (2007). Adrenomedullin and adrenomedullin-binding protein-1 downregulate inflammatory cytokines and attenuate tissue injury after gut ischemia-reperfusion. Surgery.

[9] Brell B, Hippenstiel S, David I (2005). Adrenomedullin treatment abolishes ileal mucosal hypoperfusion induced by Staphylococcus aureus alpha-toxin - an intravital microscopic study on an isolated rat ileum. Crit Care Med.

[10] Brell B, Temmesfeld-Wollbruck B, Altzschner I (2005). Adrenomedullin reduces Staphylococcus aureus alpha-toxin-induced rat ileum microcirculatory damage. Crit Care Med.

[11] Vigue B, Leblanc PE, Moati F (2016). Mid-regional pro-adrenomedullin (MR-proADM), a marker of positive fluid balance in critically ill patients: results of the ENVOL study. Crit Care.

[12] Tyagi A, Sethi AK, Girotra G, Mohta M (2009). The microcirculation in sepsis. Indian J Anaesth.

[13] Hernandez G, Bruhn A, Ince C (2013). Microcirculation in sepsis: new perspectives. Curr Vasc Pharmacol.

[14] Gille J, Ostermann H, Dragu A, Sablotzki A (2017). MR-proADM: A new biomarker for early diagnosis of sepsis in burned patients. J Burn Care Res.

[15] Bustamante A, García-Berrocoso T, Penalba A (2017). Sepsis biomarkers reprofiling to predict stroke-associated infections. J Neuroimmunol.

[16] Decker SO, Sigl A, Grumaz C (2017). Immune-response patterns and next generation sequencing diagnostics for the detection of mycoses in patients with septic shock - results of a combined clinical and experimental investigation. Int J Mol Sci.

[17] Albrich WC, Dusemund F, Ruegger K (2011). Enhancement of CURB65 score with proadrenomedullin (CURB65-A) for outcome prediction in lower respiratory tract infections: derivation of a clinical algorithm. BMC Infect Dis.

[18] Albrich WC, Ruegger K, Dusemund F (2011). Optimised patient transfer using an innovative multidisciplinary assessment in Kanton Aargau (OPTIMA I): an observational survey in lower respiratory tract infections. Swiss Med Wkly.

[19] Albrich WC, Ruegger K, Dusemund F (2013). Biomarker-enhanced triage in respiratory infections: a proof-of-concept feasibility trial. Eur Respir J.

[20] Riera J, Senna A, Cubero M (2016). Primary graft dysfunction and mortality following lung transplantation: a role for proadrenomedullin plasma levels. Am J Transplant.

[21] Schoe A, Schippers EF, Struck J (2015). Postoperative pro-adrenomedullin levels predict mortality in thoracic surgery patients: comparison with Acute Physiology and Chronic Health Evaluation IV Score. Crit Care Med.

[22] Bloos F, Trips E, Nierhaus A (2016). Effect of sodium selenite administration and procalcitonin-guided therapy on mortality in patients with severe sepsis or septic shock: a randomized clinical trial. JAMA Intern Med.

[23] Bone RC, Balk RA, Cerra FB (1992). Definitions for sepsis and organ failure and guidelines for the use of innovative therapies in sepsis. The ACCP/SCCM Consensus Conference Committee. American College of Chest Physicians/Society of Critical Care Medicine. Chest.

[24] Pencina MJ, D'Agostino RB, Steyerberg EW (2011). Extensions of net reclassification improvement calculations to measure usefulness of new biomarkers. Stat Med.

[25] Vincent JL, Marshall JC, Namendys-Silva SA (2014). Assessment of the worldwide burden of critical illness: the intensive care over nations (ICON) audit. Lancet Respir Med.

[26] Bloos F, Ruddel H, Thomas-Ruddel D (2017). Effect of a multifaceted educational intervention for anti-infectious measures on sepsis mortality: a cluster randomized trial. Intensive Care Med.

[27] Andriolo BN, Andriolo RB, Salomao R, Atallah AN (2017). Effectiveness and safety of procalcitonin evaluation for reducing mortality in adults with sepsis, severe sepsis or septic shock. Cochrane Database Syst Rev.

[28] Gonzalez-Rey E, Chorny A, Varela N, Robledo G, Delgado M (2006). Urocortin and adrenomedullin prevent lethal endotoxemia by down-regulating the inflammatory response. Am J Pathol.

[29] Temmesfeld-Wollbruck B, Hocke AC, Suttorp N, Hippenstiel S (2007). Adrenomedullin and endothelial barrier function. Thromb Haemost.

[30] Enguix-Armada A, Escobar-Conesa R, La Torre AG, De La Torre-Prados MV (2016). Usefulness of several biomarkers in the management of septic patients: C-reactive protein, procalcitonin, presepsin and mid-regional pro-adrenomedullin. Clin Chem Lab Med.

[31] Andaluz-Ojeda D, Cicuendez R, Calvo D (2015). Sustained value of proadrenomedullin as mortality predictor in severe sepsis. J Inf Secur.

[32] Christ-Crain M, Morgenthaler NG, Struck J, Harbarth S, Bergmann A, Muller B (2005). Mid-regional pro-adrenomedullin as a prognostic marker in sepsis: an observational study. Crit Care.

[33] Suberviola B, Castellanos-Ortega A, Ruiz Ruiz A, Lopez-Hoyos M, Santibanez M (2013). Hospital mortality prognostication in sepsis using the new biomarkers suPAR and proADM in a single determination on ICU admission. Intensive Care Med.

[34] Andaluz-Ojeda D, Nguyen HB, Meunier-Beillard N (2017). Superior accuracy of mid-regional proadrenomedullin for mortality prediction in sepsis with varying levels of illness severity. Ann Intensive Care.

[35] Kopterides P, Siempos II, Tsangaris I, Tsantes A, Armaganidis A (2010). Procalcitonin-guided algorithms of antibiotic therapy in the intensive care unit: a systematic review and meta-analysis of randomized controlled trials. Crit Care Med.

[36] de Jong E, van Oers JA, Beishuizen A (2016). Efficacy and safety of procalcitonin guidance in reducing the duration of antibiotic treatment in critically ill patients: a randomised, controlled, open-label trial. Lancet Infect Dis.

[37] Kip MM, Kusters R, IJzerman MJ, Steuten LM (2015). A PCT algorithm for discontinuation of antibiotic therapy is a cost-effective way to reduce antibiotic exposure in adult intensive care patients with sepsis. J Med Econ.

[38] Wilke MH, Grube RF, Bodmann KF (2011). The use of a standardized PCT-algorithm reduces costs in intensive care in septic patients - a DRG-based simulation model. Eur J Med Res.

[39] Debiane L, Hachem RY, Al Wohoush I (2014). The utility of proadrenomedullin and procalcitonin in comparison to C-reactive protein as predictors of sepsis and bloodstream infections in critically ill patients with cancer. Crit Care Med.

[40] Haubitz S, Mueller B, Schuetz P (2013). Streamlining antibiotic therapy with procalcitonin protocols: consensus and controversies. Expert Rev Respir Med.

[41] Garrouste-Orgeas M, Montuclard L, Timsit JF, Misset B, Christias M, Carlet J (2003). Triaging patients to the ICU: a pilot study of factors influencing admission decisions and patient outcomes. Intensive Care Med.

[42] Johnson DW, Schmidt UH, Bittner EA, Christensen B, Levi R, Pino RM (2013). Delay of transfer from the intensive care unit: a prospective observational study of incidence, causes, and financial impact. Crit Care.

[43] Pereira JM, Azevedo A, Basilio C, Sousa-Dias C, Mergulhao P, Paiva JA (2016). Mid-regional proadrenomedullin: an early marker of response in critically ill patients with severe community-acquired pneumonia?. Rev Port Pneumol.

[44] Miguez C, Tomatis Souverbielle C, Haro A (2016). Evaluation of proadrenomedullin as a diagnostic or prognostic biomarker of acute appendicitis in children. Am J Emerg Med.

