# Supplementary material for: The use of mid-regional proadrenomedullin to identify disease severity and treatment response to sepsis - a secondary analysis of a large randomised controlled trial
Source: Crit Care. 2018 Mar 21;22:79. doi: 10.1186/s13054-018-2001-5 (PMC5863464; doi:10.1186/s13054-018-2001-5)
Supplement: Supplementary file 1 — Supplementary results. (PDF 5376 kb) [file 13054_2018_2001_MOESM1_ESM.pdf]

Submission to *Critical Care*

Additional File 1

The use of mid-regional proadrenomedullin to identify disease severity and treatment response to sepsis - a secondary analysis of a large randomized controlled trial

Short title: The use of mid-regional proadrenomedullin to identify sepsis severity

Gunnar Elke, M.D.[1]; Frank Bloos, M.D. Ph.D.[2,3]; Darius Cameron Wilson, Ph.D.[4]; Frank M. Brunkhorst, M.D.[2,3]; Josef Briegel, M.D.[5]; Konrad Reinhart, M.D.[2,3]; Markus Loeffler, M.D.[6]; Stefan Kluge, M.D.[7]; Axel Nierhaus, M.D. EDIC[7]; Ulrich Jaschinski, M.D.[8]; Onnen Moerer, M.D.[9]; Andreas Weyland, M.D.[10]; Patrick Meybohm, M.D.[11] for the SepNet Critical Care Trials Group

1 Dept. of Anaesthesiology and Intensive Care Medicine, University Medical Center Schleswig-Holstein, Campus Kiel, Germany
2 Dept. of Anesthesiology and Intensive Care Medicine, Jena University Hospital, Germany
3 Center for Sepsis Control & Care (CSCC), Jena University Hospital, Germany
4 B·R·A·H·M·S GmbH, Hennigsdorf, Germany
5 Dept. of Anaesthesiology, University Hospital Munich, Germany
6 Clinical Trial Centre Leipzig, University of Leipzig, Germany
7 Dept. of Intensive Care Medicine, University Hospital Hamburg-Eppendorf, Germany
8 Dept. of Anaesthesiology and Surgical Intensive Care Medicine, Hospital Augsburg, Germany
9 Dept. of Anaesthesiology, University Hospital Göttingen, Germany
10 Dept. of Anaesthesiology, Hospital Oldenburg, Germany
11 Dept. of Anaesthesiology , Intensive Care Medicine and Pain Therapy, University Hospital Frankfurt, Germany

Corresponding author
Gunnar Elke, M.D.
Department of Anaesthesiology and Intensive Care Medicine
University Medical Center Schleswig-Holstein, Campus Kiel
Arnold-Heller-Str. 3 Haus 12
24105 Kiel, Germany
Tel: +49 431 500 20780
Fax: +49 431 500 20804
E-mail: gunnar.elke@uksh.de
ORCID: 0000-0002-4948-1605

CONTENTS

1. Supplementary Results..3

1.1	Influence of infectious origin and microbial species on biomarker performance
1.2	Correlation of biomarkers and clinical scores with SOFA at baseline and day 1
1.3	Identification of high risk patients
1.4	Additional prognostic value of MR-proADM measurements to baseline concentrations

2. Supplementary Tables..4

Table S1	Survival analysis for the addition of MR-proADM to baseline biomarkers or scores
Table S2	AUROC analysis for the addition of MR-proADM to baseline biomarkers or scores
Table S3	Net reclassification improvement analysis for baseline MR-proADM and biomarker/score combinations
Table S4	Survival analysis for MR-proADM within different organ dysfunction severity groups when combined with baseline biomarkers or scores
Table S5	Characterisitics of MR-proADM cut-offs at baseline
Table S6	SOFA and MR-proADM disease severity groups for 28 day mortality
Table S7	SAPS II and MR-proADM disease severity groups for 28 day mortality
Table S8	APACHE II and MR-proADM disease severity groups for 28 day mortality
Table S9	Lactate and MR-proADM disease severity groups for 28 day mortality
Table S10	Biomarker and SOFA association with 28 day mortality at days 1, 4, 7 and 10
Table S11	Disease severity groups and corresponding mortality rates throughout ICU treatment
Table S12	Characterisitics of low severity MR-proADM cut-offs at days 1, 4, 7 and 10
Table S13	28 day mortality relative risk ratios for continuously maintained biomarker and score values
Table S14	7 day, ICU and Hospital mortality rates following PCT and MR-proADM kinetics between baseline and day 1
Table S15	ICU and Hospital mortality rates following PCT and MR-proADM kinetics between baseline and day 4
Table S16	Baseline biomarker and clinical score correlation with SOFA at baseline and SOFA at day 1
Table S17	Baseline MR-proADM correlations with SOFA subscores at baseline and on day 1
Table S18	Biomarker correlations with SOFA scores throughout ICU treatment
Table S19	Time dependent Cox regressions for single and cumulative additions of MR-proADM

3. Supplementary Figures..15

Figure S1	28 and 90 day mortality rates following PCT and MR-proADM kinetics between baseline and day 4
Figure S2	Influence of infectious origin on 28 day mortality prediction
Figure S3	Influence of microbial species on 28 day mortality prediction
Figure S4	Influence of mode of ICU entry on 28 day mortality prediction

1. Supplementary Results

1.1 Influence of infectious origin and microbial species on biomarker performance

Infections originating from a single focus were found in 836 patients (77.7%), with pneumological (N = 324; 30.1%), intra-abdominal (N = 252; 23.4%), urogenital (N = 57; 5.3%) and bone/soft tissue (N = 50; 4.6%) origins most prevalent. Corresponding mortality rates were 26.5%, 24.6%, 22.8% and 28.0%, respectively. Multiple origins of infection were found in 240 (22.3%) patients. MR-proADM showed the strongest association with mortality in patients with solely pneumological and intra-abdominal infections, as well as in patients with solely Gram positive infections, irrespective of the infectious origin (**Figure S2-3**).

When patients were grouped according to operative emergency, non-operative emergency and elective surgery history resulting in admission to the ICU, MR-proADM provided the strongest and most balanced association with 28 day mortality across all groups (**Figure S4**).

1.2 Correlation of biomarkers and clinical scores with SOFA at baseline and day 1

MR-proADM had the highest correlation of all biomarkers with the SOFA score at baseline. This correlation was significantly increased when baseline MR-proADM values were correlated with day 1 SOFA scores. The greatest correlation could be found between MR-proADM and SOFA on day 10, with differences between individual SOFA subscores found throughout (**Table S16-18**).

1.3 Identification of high risk patients

The additional value of MR-proADM concentrations in the identification of high risk patients with low/intermediate SAPS II or APACHE II values could also be demonstrated. 124 patients (12.0%) were found to have high both MR-proADM concentrations and low/intermediate SAPS II values, resulting in an increased 28 and 90 day mortality rate of 54.8% and 65.6%, compared to a 28 and 90 day mortality rate of 19.7% and 30.0% in the remaining low/intermediate SAPS II population. This was also found to be the case when the APACHE II score was used, with the identification of 109 (10.6%) patients with high MR-proADM and low/intermediate APACHE II values, resulting in a 28 and 90 day mortality rate of 56.9% and 66.7%, as opposed to a 28 and 90 day mortality rate of 19.5% and 30.3% in the remaining low/intermediate APACHE II population.

1.4 Additional prognostic value of MR-proADM measurements to baseline concentrations

Time-dependent Cox regression analysis indicated that the earliest significant additional increase in prognostic information to MR-proADM baseline values could be observed on day 1, with subsequent or cumulative measurements resulting in significantly stronger associations with 28 day mortality (**Table S19**).

2. Supplementary Tables

Table S1. Survival analysis for the addition of MR-proADM to baseline biomarkers or scores

				Bivariate Cox regression			Added value		Multivariate Cox regression			Added value	
	Biomarker or clinical score	Patients (N)	Mortality (N)	LR χ^2	C-index	HR IQR [95% CI]	LR χ^2	p-value	LR χ^2	C-index	HR IQR [95% CI]	LR χ^2	p-value
7 day mortality	PCT	1037	131	76.5	0.72	4.0 [2.9 - 5.6]	66.8	<0.001	86.2	0.73	4.2 [2.9 - 6.1]	57.8	<0.001
	CRP	904	108	56.9	0.71	3.2 [2.3 - 4.3]	55.0	<0.001	67.7	0.73	3.3 [2.3 - 4.7]	49.4	<0.001
	Lactate	1029	131	112.5	0.75	2.3 [1.7 - 3.1]	28.1	<0.001	125.1	0.76	2.4 [1.7 - 3.3]	26.4	<0.001
	SOFA	1014	126	77.8	0.72	3.3 [2.3 - 4.6]	53.5	<0.001	86.9	0.74	3.3 [2.3 - 4.7]	46.6	<0.001
	SAPS II	1037	131	83.1	0.73	2.8 [2.0 - 3.7]	48.1	<0.001	93.5	0.74	2.9 [2.1 - 4.0]	46.7	<0.001
	APACHE II	1037	131	73.3	0.71	3.0 [2.2 - 4.1]	50.9	<0.001	84.5	0.73	3.1 [2.2 - 4.2]	48.6	<0.001
28 day mortality	PCT	1030	275	163.0	0.73	4.3 [3.4 - 5.5]	150.7	<0.001	174.9	0.73	3.9 [3.0 - 5.1]	105.0	<0.001
	CRP	898	239	114.4	0.70	3.0 [2.5 - 3.8]	114.2	<0.001	132.4	0.72	2.8 [2.2 - 3.6]	80.5	<0.001
	Lactate	1022	275	163.8	0.72	2.7 [2.2 - 3.3]	85.9	<0.001	184.5	0.73	2.5 [2.0 - 3.1]	61.4	<0.001
	SOFA	1007	268	150.6	0.72	3.1 [2.5 - 3.9]	104.1	<0.001	169.9	0.73	2.8 [2.2 - 3.6]	74.4	<0.001
	SAPS II	1030	275	163.4	0.72	2.7 [2.2 - 3.3]	97.1	<0.001	176.5	0.73	2.6 [2.1 - 3.3]	79.1	<0.001
	APACHE II	1030	275	153.6	0.72	2.7 [2.2 - 3.4]	88.8	<0.001	169.1	0.73	2.6 [2.1 - 3.3]	74.1	<0.001
90 day mortality	PCT	1000	379	170.8	0.70	3.6 [3.0 - 4.4]	159.0	<0.001	208.2	0.71	3.1 [2.5 - 3.9]	94.8	<0.001
	CRP	872	331	116.0	0.68	2.6 [2.2 - 3.1]	116.0	<0.001	160.3	0.70	2.3 [1.9 - 2.8]	68.8	<0.001
	Lactate	993	379	169.4	0.69	2.3 [1.9 - 2.7]	86.6	<0.001	217.5	0.71	2.0 [1.7 - 2.4]	50.2	<0.001
	SOFA	977	368	151.0	0.69	2.6 [2.1 - 3.1]	103.1	<0.001	200.6	0.71	2.2 [1.8 - 2.7]	59.9	<0.001
	SAPS II	1000	379	173.7	0.70	2.3 [1.9 - 2.7]	94.7	<0.001	208.4	0.71	2.2 [1.8 - 2.6]	67.6	<0.001
	APACHE II	1000	379	165.0	0.70	2.3 [1.9 - 2.7]	83.3	<0.001	202.9	0.71	2.1 [1.8 - 2.6]	62.5	<0.001
ICU mortality	PCT	1023	264	149.5	0.75	5.7 [4.1 - 7.9]	131.4	<0.001	165.3	0.76	4.9 [3.5 - 7.0]	92.6	<0.001
	CRP	889	226	104.6	0.72	3.7 [2.8 - 4.8]	102.5	<0.001	127.4	0.74	3.4 [2.5 - 4.6]	75.6	<0.001
	Lactate	1015	264	153.5	0.74	3.2 [2.4 - 4.2]	78.9	<0.001	175.6	0.76	2.9 [2.2 - 3.9]	57.5	<0.001
	SOFA	1000	257	140.7	0.74	3.6 [2.7 - 4.8]	91.8	<0.001	163.8	0.76	3.2 [2.4 - 4.4]	65.8	<0.001
	SAPS II	1023	264	152.5	0.75	3.4 [2.6 - 4.4]	94.4	<0.001	169.2	0.76	3.3 [2.5 - 4.3]	77.7	<0.001
	APACHE II	1023	264	148.2	0.74	3.3 [2.5 - 4.4]	87.9	<0.001	165.7	0.76	3.3 [2.5 - 4.3]	75.6	<0.001
Hospital mortality	PCT	980	323	174.7	0.76	6.4 [4.6 - 8.8]	159.5	<0.001	198.9	0.77	5.2 [3.6 - 7.3]	103.2	<0.001
	CRP	852	283	117.9	0.72	3.7 [2.9 - 4.8]	117.3	<0.001	150.1	0.75	3.3 [2.5 - 4.3]	77.7	<0.001
	Lactate	972	323	167.4	0.75	3.3 [2.5 - 4.3]	89.2	<0.001	202.5	0.76	2.8 [2.1 - 3.8]	57.6	<0.001
	SOFA	957	314	155.5	0.74	3.9 [3.0 - 5.2]	113.7	<0.001	191.3	0.76	3.4 [2.5 - 4.5]	74.6	<0.001
	SAPS II	980	323	165.8	0.75	3.5 [2.7 - 4.5]	107.7	<0.001	194.2	0.76	3.2 [2.4 - 4.2]	81.3	<0.001
	APACHE II	980	323	169.7	0.75	3.3 [2.6 - 4.3]	95.4	<0.001	197.2	0.76	3.1 [2.4 - 4.1]	75.1	<0.001

HR IQR [95% CI] indicates the hazard ratio for MR-proADM in each bivariate or multivariate model. 2 degrees of freedom in each bivariate model, compared to 11 in each multivariate model. Additional combination data not shown.

APACHE II: Acute Physiological and Chronic Health Evaluation II score; *CI*: Confidence Interval; *CRP*: C-reactive protein; *HR*: Hazard Ratio; *IQR*: Interquartile range; *MR-proADM*: Mid-regional proadrenomedullin; *N*: Number; *PCT*: Procalcitonin; *SOFA*: Sequential Organ Failure Assessment score; *SAPS II*: Simplified Acute Physiological II score.

Table S2. **AUROC analysis for the addition of MR-proADM to baseline biomarkers or scores**

	Biomarker or score	AUROC [95% CI]	Biomarker or score + MR-proADM	AUROC [95% CI]
**7 day mortality**	**MR-proADM**	0.72 [0.67 - 0.77]		
	**PCT**	0.58 [0.53 - 0.63]	**PCT**	0.73 [0.68 - 0.78]
	**CRP**	0.55 [0.49 - 0.61]	**CRP**	0.72 [0.66 - 0.77]
	**Lactate**	0.72 [0.67 - 0.77]	**Lactate**	0.75 [0.71 - 0.80]
	**SOFA**	0.64 [0.59 - 0.68]	**SOFA**	0.73 [0.68 - 0.78]
	**SAPS II**	0.66 [0.62 - 0.71]	**SAPS II**	0.73 [0.68 - 0.78]
	**APACHE II**	0.63 [0.58 - 0.68]	**APACHE II**	0.72 [0.67 - 0.77]
**28 day mortality**	**MR-proADM**	0.73 [0.70 - 0.77]		
	**PCT**	0.56 [0.52 – 0.60]	**PCT**	0.76 [0.72 - 0.79]
	**CRP**	0.49 [0.45 - 0.53]	**CRP**	0.73 [0.69 - 0.76]
	**Lactate**	0.65 [0.61 - 0.69]	**Lactate**	0.74 [0.70 - 0.77]
	**SOFA**	0.64 [0.60 - 0.68]	**SOFA**	0.74 [0.71 - 0.78]
	**SAPS II**	0.67 [0.63 - 0.70]	**SAPS II**	0.75 [0.72 - 0.78]
	**APACHE II**	0.67 [0.64 - 0.70]	**APACHE II**	0.75 [0.71 - 0.78]
**90 day mortality**	**MR-proADM**	0.71 [0.68 - 0.74]		
	**PCT**	0.55 [0.51 - 0.59]	**PCT**	0.73 [0.70 - 0.77]
	**CRP**	0.50 [0.46 - 0.53]	**CRP**	0.70 [0.67 - 0.74]
	**Lactate**	0.64 [0.60 - 0.67]	**Lactate**	0.72 [0.68 - 0.75]
	**SOFA**	0.63 [0.59 - 0.66]	**SOFA**	0.72 [0.68 - 0.75]
	**SAPS II**	0.66 [0.63 - 0.69]	**SAPS II**	0.73 [0.70 - 0.76]
	**APACHE II**	0.67 [0.64 - 0.70]	**APACHE II**	0.73 [0.70 - 0.76]
**ICU mortality**	**MR-proADM**	0.73 [0.70 - 0.77]		
	**PCT**	0.58 [0.54 - 0.62]	**PCT**	0.75 [0.71 - 0.78]
	**CRP**	0.54 [0.49 - 0.58]	**CRP**	0.72 [0.68 - 0.76]
	**Lactate**	0.66 [0.62 - 0.70]	**Lactate**	0.74 [0.71 - 0.78]
	**SOFA**	0.64 [0.60 - 0.68]	**SOFA**	0.75 [0.70 - 0.77]
	**SAPS II**	0.65 [0.61 - 0.69]	**SAPS II**	0.75 [0.71 - 0.78]
	**APACHE II**	0.66 [0.62 - 0.69]	**APACHE II**	0.74 [0.71 - 0.78]
**Hospital mortality**	**MR-proADM**	0.74 [0.70 - 0.77]		
	**PCT**	0.57 [0.53 - 0.61]	**PCT**	0.76 [0.72 - 0.79]
	**CRP**	0.52 [0.48 - 0.56]	**CRP**	0.72 [0.69 - 0.76]
	**Lactate**	0.66 [0.62 - 0.67]	**Lactate**	0.75 [0.71 - 0.78]
	**SOFA**	0.63 [0.59 - 0.66]	**SOFA**	0.74 [0.71 - 0.77]
	**SAPS II**	0.65 [0.61 - 0.68]	**SAPS II**	0.75 [0.71 - 0.78]
	**APACHE II**	0.67 [0.63 - 0.70]	**APACHE II**	0.75 [0.72 - 0.78]

AUROC: Area Under the Receiver Operating Characteristic; *APACHE II*: Acute Physiological and Chronic Health Evaluation II score; *CI*: Confidence Interval; *CRP*: C-reactive protein; *MR-proADM*: Mid-regional proadrenomedullin; *PCT*: Procalcitonin; *SAPS* II: Simplified Acute Physiological II score; *SOFA*: Sequential Organ Failure Assessment score

Table S3. Net reclassification improvement analysis for baseline MR-proADM and biomarker/score combinations

	Biomarker or score	NRI total population [95% CI]	NRI survivors [95% CI]	NRI non-survivors [95% CI]
**28 day mortality**	**PCT**	0.79 [0.62 - 0.91]	0.39 [0.29 - 0.46]	0.40 [0.30 - 0.48]
	**CRP**	0.67 [0.53 - 0.81]	0.30 [0.23 - 0.38]	0.37 [0.27 - 0.45]
	**Lactate**	0.66 [0.52 - 0.80]	0.31 [0.23 - 0.38]	0.35 [0.27 - 0.43]
	**SOFA**	0.63 [0.48 - 0.76]	0.29 [0.22 - 0.36]	0.34 [0.24 - 0.42]
	**SAPS II**	0.62 [0.48 - 0.74]	0.25 [0.19 - 0.33]	0.37 [0.26 - 0.43]
	**APACHE II**	0.54 [0.42 - 0.69]	0.23 [0.15 - 0.32]	0.31 [0.24 - 0.40]
**90 day mortality**	**PCT**	0.73 [0.61 - 0.84]	0.36 [0.29 - 0.43]	0.37 [0.29 - 0.43]
	**CRP**	0.61 [0.47 - 0.74]	0.31 [0.23 - 0.37]	0.30 [0.22 - 0.38]
	**Lactate**	0.58 [0.45 - 0.71]	0.29 [0.20 - 0.36]	0.29 [0.22 - 0.38]
	**SOFA**	0.56 [0.43 - 0.69]	0.27 [0.20 - 0.35]	0.29 [0.22 - 0.36]
	**SAPS II**	0.55 [0.42 - 0.67]	0.25 [0.17 - 0.32]	0.30 [0.23 - 0.37]
	**APACHE II**	0.50 [0.37 - 0.61]	0.22 [0.14 - 0.28]	0.28 [0.21 - 0.36]

APACHE II: Acute Physiological and Chronic Health Evaluation II score; *CI*: Confidence Interval; *CRP*: C-reactive protein; *MR-proADM*: Mid-regional proadrenomedullin; *NRI*: Net Reclassification Improvement; *PCT*: Procalcitonin; *SAPS II*: Simplified Acute Physiological II score; *SOFA*: Sequential Organ Failure Assessment score

Table S4. Survival analysis for MR-proADM within different organ dysfunction severity groups when combined with baseline biomarkers or scores

				Bivariate Cox regression				Multivariate Cox regression			
	Biomarker or clinical score	Patients (N)	Mortality (N)	LR χ^2	C-index	HR IQR [95% CI]	p-value	LR χ^2	C-index	HR IQR [95% CI]	p-value
SOFA ≤7	PCT	232	32	30.0	0.75	5.3 [2.8 - 10.1]	<0.001	41.8	0.78	5.0 [2.3 - 10.8]	<0.001
	CRP	204	29	20.1	0.71	3.1 [1.8 - 5.3]	<0.001	30.5	0.75	2.7 [1.4 - 5.0]	0.001
	Lactate	229	32	25.1	0.72	3.5 [2.0 - 5.9]	<0.001	37.2	0.77	3.1 [1.7 - 5.7]	<0.001
	SOFA	232	32	27.3	0.73	3.9 [2.3 - 6.7]	<0.001	40.4	0.78	3.5 [1.9 - 6.5]	<0.001
	SAPS II	232	32	28.9	0.74	3.2 [1.9 - 5.4]	<0.001	38.4	0.78	3.1 [1.7 - 5.5]	<0.001
	APACHE II	232	32	34.2	0.77	2.9 [1.7 - 4.9]	<0.001	41.4	0.79	3.0 [1.7 - 5.5]	<0.001
SOFA 8 - 13	PCT	620	172	90.4	0.72	3.8 [2.8 - 5.0]	<0.001	98.0	0.72	3.2 [2.3 - 4.4]	<0.001
	CRP	544	153	63.1	0.69	2.6 [2.0 - 3.3]	<0.001	78.6	0.71	2.4 [1.7 - 2.9]	<0.001
	Lactate	617	172	81.4	0.70	2.4 [1.9 - 3.1]	<0.001	97.0	0.72	2.1 [1.6 - 2.7]	<0.001
	SOFA	620	172	76.2	0.70	2.6 [2.0 - 3.2]	<0.001	90.7	0.72	2.3 [1.8 - 2.9]	<0.001
	SAPS II	620	172	87.2	0.71	2.4 [1.9 - 3.1]	<0.001	97.2	0.72	2.3 [1.8 - 2.9]	<0.001
	APACHE II	620	172	79.0	0.70	2.5 [1.9 - 3.1]	<0.001	90.9	0.72	2.3 [1.8 - 2.9]	<0.001
SOFA ≥14	PCT	155	64	16.3	0.66	2.2 [1.5 - 3.2]	0.001	27.1	0.69	2.4 [1.5 - 3.9]	0.001
	CRP	134	52	13.4	0.65	1.9 [1.3 - 2.9]	0.001	26.9	0.70	2.1 [1.3 - 3.3]	0.001
	Lactate	155	64	28.9	0.69	1.7 [1.1 - 2.5]	0.006	38.1	0.71	1.8 [1.1 - 2.8]	0.007
	SOFA	155	64	15.3	0.65	2.0 [1.3 - 2.9]	<0.001	26.7	0.69	2.1 [1.3 - 3.2]	<0.001
	SAPS II	155	64	17.0	0.65	2.1 [1.4 - 3.1]	<0.001	26.2	0.69	2.2 [1.4 - 3.3]	<0.001
	APACHE II	155	64	15.1	0.64	2.0 [1.3 - 2.9]	<0.001	25.7	0.69	2.1 [1.4 - 3.3]	<0.001

HR IQR [95% CI] indicates the hazard ratio for MR-proADM in each bivariate or multivariate model. *APACHE II*: Acute Physiological and Chronic Health Evaluation II score; *CI*: Confidence Interval; *CRP*: C-reactive protein; *HR*: Hazard Ratio; *IQR*: Interquartile range; *MR-proADM*: Mid-regional proadrenomedullin; *N*: Number; *PCT*: Procalcitonin; *SAPS II*: Simplified Acute Physiological II score; *SOFA*: Sequential Organ Failure Assessment score

Table S5. Characterisitics of MR-proADM cut-offs at baseline

MR-proADM cut-off (nmol/L)	Sensitivity	Specificity	PPV	NPV	LR+	LR-
2.75	0.90 [0.86 - 0.93]	0.32 [0.29 - 0.36]	0.33 [0.29 - 0.36]	0.90 [0.85 - 0.93]	1.32 [1.24 - 1.41]	0.32 [0.22 - 0.46]
10.9	0.33 [0.27 - 0.38]	0.90 [0.88 - 0.92]	0.55 [0.47 - 0.62]	0.79 [0.76 - 0.81]	3.29 [2.51 - 4.33]	0.75 [0.69 - 0.81]

LR+: Positive likelihood ratio; *LR-*: Negative likelihood ratio; *MR-proADM*: Mid-regional proadrenomedullin; *NPV*: Negative predictive value; *PPV*: Positive predictive value

Table S6. **SOFA and MR-proADM disease severity groups for 28 day mortality**

		**SOFA severity groups**		
**MR-proADM severity groups**		**Low severity<br>(≤7 points)<br>N = 232, 13.8% mortality**	**Intermediate severity<br>(≥8 points ≤13)<br>N = 620, 27.7% mortality**	**High severity<br>(≥14 points)<br>N = 155, 41.3% mortality**
	**Low severity<br>(≤2.7 nmol/L)<br>N = 265, 9.8% mortality**	N = 111 (41.9%)<br>7.2% mortality	N = 139 (52.8%)<br>10.8% mortality	N = 15 (5.7%)<br>20.0% mortality
	**Intermediate severity<br>(>2.7 nmol/L ≤10.9)<br>N = 581, 26.2% mortality**	N = 114 (19.6%)<br>15.8% mortality	N = 394 (68.0%)<br>27.7% mortality	N = 73 (12.6%)<br>34.2% mortality
	**High severity<br>(>10.9 nmol/L)<br>N = 161, 55.9% mortality**	N = 7 (4.3%)<br>85.7% mortality	N = 87 (53.4%)<br>55.2% mortality	N = 67 (41.6%)<br>53.7% mortality

MR-proADM: Mid-regional proadrenomedullin; *N*: Number; *SOFA*: Sequential Organ Failure Assessment score

Table S7. **SAPS II and MR-proADM disease severity groups for 28 day mortality**

		**SAPS II severity groups**		
**MR-proADM severity groups**		**Low severity<br>(≤53 points)<br>N = 235, 11.5% mortality**	**Intermediate severity<br>(≥54 points ≤79)<br>N = 656, 29.3% mortality**	**High severity<br>(≥80 points)<br>N = 139, 40.3% mortality**
	**Low severity<br>(≤2.7 nmol/L)<br>N = 271, 10.3% mortality**	N = 108 (39.9%)<br>7.4% mortality	N = 143 (52.8%)<br>11.2% mortality	N = 20 (7.4%)<br>20.0% mortality
	**Intermediate severity<br>(>2.7 nmol/L ≤10.9)<br>N = 594, 26.4% mortality**	N = 118 (19.9%)<br>13.6% morality	N = 398 (67.0%)<br>27.9% mortality	N = 78 (13.1%)<br>38.5% mortality
	**High severity<br>(>10.9 nmol/L)<br>N = 165, 54.5% mortality**	N = 9 (5.5%)<br>33.3% mortality	N = 115 (69.7%)<br>56.5% mortality	N = 41 (24.8%)<br>53.7% mortality

MR-proADM: Mid-regional proadrenomedullin; *N*: Number; *SAPS II*: Simplified Acute Physiological II score

Table S8. **APACHE II and MR-proADM disease severity groups for 28 day mortality**

		**APACHE II severity groups**		
**MR-proADM severity groups**		**Low severity<br>(≤19 points)<br>N = 287, 11.5% mortality**	**Intermediate severity<br>(≥20 points ≤32)<br>N = 591, 30.3% mortality**	**High severity<br>(≥33 points)<br>N = 152, 41.4% mortality**
	**Low severity<br>(≤2.7 nmol/L)<br>N = 271, 10.3% mortality**	N = 122 (45.0%)<br>7.4% mortality	N = 137 (50.6%)<br>10.9% mortality	N = 12 (4.4%)<br>33.3% mortality
	**Intermediate severity<br>(>2.7 nmol/L ≤10.9)<br>N = 594, 26.4% mortality**	N = 154 (25.9%)<br>12.3% mortality	N = 356 (59.9%)<br>30.1% mortality	N = 84 (14.1%)<br>36.9% mortality
	**High severity<br>(>10.9 nmol/L)<br>N = 165, 54.5% mortality**	N = 11 (6.7%)<br>45.5% mortality	N = 98 (59.4%)<br>58.2% mortality	N = 56 (33.9%)<br>50.0% mortality

APACHE II: Acute Physiological and Chronic Health Evaluation II score; *MR-proADM*: Mid-regional proadrenomedullin; *N*: Number

Table S9. **Lactate and MR-proADM disease severity groups for 28 day mortality**

		Lactate severity groups		
MR-proADM severity groups		**Low severity (≤1.4 mmol/L) N = 196, 15.8% mortality**	**Intermediate severity (>1.4 mmol/L ≤6.4) N = 668, 24.1% mortality**	**High severity (>6.4 mmol/L) N = 158, 52.5% mortality**
	**Low severity (≤2.7 nmol/L) N = 267, 10.5% mortality**	N = 99 (37.1%) 8.1% mortality	N = 154 (57.7%) 9.1% mortality	N = 14 (5.2%) 42.9% mortality
	**Intermediate severity (>2.7 nmol/L ≤10.9) N = 591, 26.6% mortality**	N = 90 (15.2%) 21.1% mortality	N = 421 (71.2%) 25.2% mortality	N = 80 (13.5%) 40.0% mortality
	**High severity (>10.9 nmol/L) N = 164, 54.9% mortality**	N = 7 (4.3%) 57.1% mortality	N = 93 (56.7%) 44.1% mortality	N = 64 (39.0%) 70.3% mortality

MR-proADM: Mid-regional proadrenomedullin; *N*: Number

Table S10. **Biomarker and SOFA association with 28 day mortality at days 1, 4, 7 and 10**

					Univariate Cox regression				Multivariate Cox regression			
	Biomarker or clinical score	Patients (N)	Mortality (N)	AUROC	LR χ^2	C-index	HR IQR [95% CI]	p-value	LR χ^2	C-index	HR IQR [95% CI]	p-value
Day 1	**MR-proADM**	993	242	0.76	152.5	0.73	3.3 [2.8 - 4.0]	<0.001	173.2	0.74	3.2 [2.6 - 4.0]	<0.001
	**PCT**	993	242	0.59	23.1	0.59	1.6 [1.3 - 2.0]	<0.001	74.6	0.65	1.6 [1.3 - 2.0]	<0.001
	**CRP**	919	226	0.54	6.2	0.54	0.9 [0.8 - 1.0]	0.013	61.2	0.65	0.9 [0.8 - 1.0]	<0.001
	**Lactate**	1041	265	0.73	206.4	0.72	2.4 [2.2 - 2.7]	<0.001	253.9	0.75	2.5 [2.2 - 2.8]	<0.001
	**SOFA**	1011	260	0.74	143.8	0.72	2.5 [2.2 - 2.9]	<0.001	192.8	0.75	2.6 [2.2 - 3.0]	<0.001
Day 4	**MR-proADM**	777	158	0.76	100.5	0.73	3.2 [2.5 - 4.0]	<0.001	123.7	0.75	3.0 [2.3 - 3.8]	<0.001
	**PCT**	777	158	0.62	22.6	0.61	1.7 [1.4 - 2.1]	<0.001	69.3	0.68	1.8 [1.4 - 2.2]	<0.001
	**CRP**	708	146	0.48	0.7	0.52	1.1 [0.9 - 1.3]	0.393	45.8	0.65	1.1 [0.9 - 1.4]	<0.001
	**Lactate**	803	166	0.69	60.6	0.68	1.8 [1.6 - 2.0]	<0.001	100.9	0.71	1.7 [1.5 - 2.0]	<0.001
	**SOFA**	767	162	0.75	111.5	0.72	3.0 [2.4 - 3.6]	<0.001	155.9	0.76	3.1 [2.5 - 3.8]	<0.001
Day 7	**MR-proADM**	630	127	0.78	93.7	0.76	3.4 [2.6 - 4.3]	<0.001	117.8	0.76	3.3 [2.5 - 4.3]	<0.001
	**PCT**	631	128	0.72	62.3	0.70	2.6 [2.1 - 3.3]	<0.001	101.6	0.74	2.7 [2.1 - 3.4]	<0.001
	**CRP**	583	121	0.56	3.5	0.55	1.3 [1.0 - 1.6]	0.061	47.1	0.67	1.3 [1.0 - 1.7]	<0.001
	**Lactate**	658	138	0.68	69.4	0.68	2.0 [1.7 - 2.3]	<0.001	112.2	0.73	2.0 [1.7 - 2.4]	<0.001
	**SOFA**	617	128	0.75	107.7	0.73	2.7 [2.3 - 3.3]	<0.001	140.2	0.77	2.8 [2.3 - 3.4]	<0.001
Day 10	**MR-proADM**	503	82	0.78	72.6	0.76	4.3 [3.0 - 6.1]	<0.001	90.9	0.78	3.8 [2.6 - 5.5]	<0.001
	**PCT**	503	82	0.75	52.0	0.74	2.8 [2.2 - 3.7]	<0.001	90.4	0.78	3.1 [2.3 - 4.2]	<0.001
	**CRP**	457	80	0.61	10.0	0.60	1.6 [1.2 - 2.2]	<0.001	51.2	0.71	1.8 [1.3 - 2.6]	<0.001
	**Lactate**	516	88	0.61	19.8	0.61	1.6 [1.3 - 2.0]	<0.001	54.7	0.70	1.6 [1.3 - 2.0]	<0.001
	**SOFA**	490	84	0.76	85.8	0.75	3.3 [2.6 - 4.3]	<0.001	107.8	0.78	3.1 [2.4 - 4.1]	<0.001

APACHE II: Acute Physiological and Chronic Health Evaluation II score; *CI*: Confidence Interval; *CRP*: C-reactive protein; *HR*: Hazard Ratio; *IQR*: Interquartile range; *MR-proADM*: Mid-regional proadrenomedullin; *N*: Number; *PCT*: Procalcitonin; *SAPS II*: Simplified Acute Physiological II score; *SOFA*: Sequential Organ Failure Assessment score

Table S11. **Disease severity groups and corresponding mortality rates throughout ICU treatment**

		Low severity patient population					High severity patient population				
	Biomarker or clinical score	Patients (N)	Mortality (N, %)	Optimal cut-off	Sensitivity	Specificity	Patients (N)	Mortality (N, %)	Optimal cut-off	Sensitivity	Specificity
Day 1	MR-proADM	304	24 (7.9%)	2.80	0.90	0.37	162	87 (53.7%)	9.5	0.36	0.90
	PCT	203	25 (12.3%)	1.02	0.90	0.24	115	40 (34.8%)	47.6	0.17	0.90
	CRP	101	32 (31.7%)	99	0.90	0.14	88	18 (4.8%)	373	0.08	0.90
	Lactate	310	33 (10.6%)	1.22	0.88	0.36	185	109 (58.9%)	3.5	0.43	0.89
	SOFA	435	49 (11.3%)	8.0	0.88	0.40	165	87 (52.7%)	14	0.33	0.90
Day 4	MR-proADM	290	16 (5.5%)	2.25	0.90	0.44	120	58 (48.3%)	7.7	0.37	0.90
	PCT	147	16 (10.9%)	0.33	0.90	0.21	87	25 (28.7%)	14.08	0.16	0.90
	CRP	65	9 (13.8%)	32.7	0.90	0.06	51	15 (29.4%)	276.5	0.06	0.90
	Lactate	124	15 (12.1%)	0.89	0.91	0.17	136	65 (47.8%)	2.15	0.39	0.89
	SOFA	213	15 (7.0%)	5.5	0.91	0.33	137	67 (48.9%)	12.75	0.41	0.88
Day 7	MR-proADM	252	14 (5.6%)	2.25	0.89	0.47	104	54 (51.9%)	6.95	0.43	0.90
	PCT	184	14 (7.6%)	0.31	0.89	0.34	85	35 (41.2%)	4.67	0.27	0.90
	CRP	62	12 (19.4%)	27.4	0.90	0.11	69	23 (37.7%)	207	0.19	0.90
	Lactate	104	15 (14.4%)	0.84	0.89	0.17	102	51 (50.0%)	2.10	0.37	0.90
	SOFA	207	16 (7.7%)	5.5	0.88	0.39	91	48 (52.7%)	12.5	0.38	0.91
Day 10	MR-proADM	213	8 (3.8%)	2.25	0.90	0.49	78	35 (44.9%)	7.45	0.43	0.90
	PCT	177	9 (5.1%)	0.30	0.89	0.40	74	32 (43.2%)	2.845	0.39	0.90
	CRP	69	8 (11.6%)	32.1	0.90	0.16	52	14 (26.9%)	204	0.18	0.90
	Lactate	47	7 (14.9%)	0.68	0.92	0.09	65	24 (36.9%)	2.15	0.27	0.90
	SOFA	116	9 (7.8%)	4.5	0.89	0.26	85	42 (49.4%)	11.5	0.50	0.89

CRP: C-reactive protein; *MR-proADM*: Mid-regional proadrenomedullin; *N*: Number; *PCT*: Procalcitonin; *SOFA*: Sequential Organ Failure Assessment score

Table S12. **Characterisitics of low severity MR-proADM cut-offs at days 1, 4, 7 and 10**

Time point	MR-proADM cut-off (nmol/L)	Sensitivity	Specificity	PPV	NPV	LR+	LR-
**Day 1**	2.80	0.90 [0.86 - 0.93]	0.37 [0.34 - 0.41]	0.32 [0.28 - 0.35]	0.92 [0.89 - 0.95]	1.44 [1.34 - 1.54]	0.26 [0.18 - 0.39]
**Day 4**	2.25	0.90 [0.84 - 0.94]	0.44 [0.40 - 0.48]	0.29 [0.25 - 0.33]	0.94 [0.91 - 0.97]	1.61 [1.48 - 1.76]	0.23 [0.14 - 0.37]
**Day 7**	2.25	0.89 [0.82 - 0.93]	0.47 [0.43 - 0.52]	0.30 [0.26 - 0.35]	0.94 [0.91 - 0.97]	1.69 [1.52 - 1.87]	0.23 [0.14 - 0.39]
**Day 10**	2.25	0.90 [0.82 - 0.95]	0.49 [0.44 - 0.53]	0.26 [0.21 - 0.31]	0.96 [0.93 - 0.98]	1.76 [1.56 - 1.98]	0.20 [0.10 - 0.39]

LR+: Positive likelihood ratio; *LR-*: Negative likelihood ratio; *MR-proADM*: Mid-regional proadrenomedullin; *NPV*: Negative predictive value; *PPV*: Positive predictive value

Table S13. **28 day mortality relative risk ratios for continuously maintained biomarker and score values**

Ventile (%)	MR-proADM cut-off (nmol/L)	MR-proADM Relative Risk Ratio [95% CI]	MR-proADM χ^2 test p-value	PCT cut-off (ng/mL)	PCT Relative Risk Ratio [95% CI]	PCT χ^2 test p-value	Lactate cut-off (mmol/L)	Lactate Relative Risk Ratio [95% CI]	Lactate χ^2 test p-value	SOFA cut-off (points)	SOFA Relative Risk Ratio [95% CI]	SOFA χ^2 test p-value
**5**	1.3	6.03 [3.52 - 10.32]	<0.001	0.25	3.44 [2.35 - 5.02]	<0.001	1.0	3.15 [2.45 - 4.05]	<0.001	5	4.99 [3.66 - 6.82]	<0.001
**10**	1.6	5.57 [3.60 - 8.60]	<0.001	0.43	3.05 [2.30 - 4.06]	<0.001	1.1	3.34 [2.64 - 4.22]	<0.001	6	4.70 [3.64 - 6.09]	<0.001
**15**	2	5.88 [4.11 - 8.41]	<0.001	0.78	3.12 [2.45 - 3.97]	<0.001	1.3	3.53 [2.88 - 4.33]	<0.001	7	4.24 [3.40 - 5.28]	<0.001
**20**	2.3	4.64 [3.45 - 6.22]	<0.001	1.20	3.00 [2.41 - 3.74]	<0.001	1.5	3.89 [3.22 - 4.70]	<0.001	7	4.24 [3.40 - 5.28]	<0.001
**25**	2.6	4.59 [3.49 - 6.03]	<0.001	1.59	3.05 [2.47 - 3.75]	<0.001	1.6	3.63 [3.04 - 4.35]	<0.001	8	3.92 [3.22 - 4.77]	<0.001
**30**	3	4.61 [3.60 - 5.92]	<0.001	2.30	2.66 [2.19 - 3.23]	<0.001	1.8	3.83 [3.23 - 4.53]	<0.001	8	3.92 [3.22 - 4.77]	<0.001
**35**	3.4	4.27 [3.41 - 5.34]	<0.001	3.16	2.60 [2.16 - 3.14]	<0.001	2.0	3.89 [3.30 - 4.60]	<0.001	9	3.64 [3.05 - 4.35]	<0.001
**40**	3.9	4.32 [3.50 - 5.33]	<0.001	4.28	2.48 [2.06 - 2.99]	<0.001	2.2	4.35 [3.72 - 5.08]	<0.001	9	3.64 [3.05 - 4.35]	<0.001
**45**	4.4	4.18 [3.43 - 5.10]	<0.001	4.53	2.48 [2.07 - 2.99]	<0.001	2.4	4.59 [3.96 - 5.33]	<0.001	9	3.64 [3.05 - 4.35]	<0.001
**50**	4.9	4.14 [3.42 - 5.01]	<0.001	7.37	2.42 [2.01 - 2.92]	<0.001	2.7	4.58 [3.97 - 5.28]	<0.001	10	3.45 [2.91 - 4.08]	<0.001
**55**	5.5	3.89 [3.24 - 4.67]	<0.001	9.22	2.38 [1.97 - 2.87]	<0.001	2.9	4.58 [3.98 - 5.27]	<0.001	10	3.45 [2.91 - 4.08]	<0.001
**60**	6.2	3.62 [3.03 - 4.32]	<0.001	11.24	2.22 [1.83 - 2.69]	<0.001	3.2	4.39 [3.82 - 5.05]	<0.001	10	3.45 [2.91 - 4.08]	<0.001
**65**	7.0	3.51 [2.96 - 4.17]	<0.001	14.71	2.17 [1.78 - 2.66]	<0.001	3.7	4.18 [3.64 - 4.81]	<0.001	11	3.22 [2.72 - 3.81]	<0.001
**70**	7.8	3.12 [2.62 - 3.71]	<0.001	18.83	2.06 [1.66 - 2.55]	<0.001	4.0	4.09 [3.56 - 4.69]	<0.001	11	3.22 [2.72 - 3.81]	<0.001
**75**	8.8	3.03 [2.55 - 3.61]	<0.001	26.77	2.14 [1.71 - 2.66]	<0.001	4.7	4.01 [3.50 - 4.60]	<0.001	12	2.97 [2.48 - 3.56]	<0.001
**80**	9.6	2.81 [2.34 - 3.38]	<0.001	37.96	2.06 [1.61 - 2.62]	<0.001	5.4	3.77 [3.26 - 4.36]	<0.001	13	2.95 [2.45 - 3.56]	<0.001
**85**	11.3	2.68 [2.19 - 3.27]	<0.001	46.27	2.05 [1.60 - 2.63]	<0.001	6.6	3.83 [3.33 - 4.40]	<0.001	13	2.95 [2.45 - 3.56]	<0.001
**90**	14.1	2.48 [1.98 - 3.10]	<0.001	59.12	1.96 [1.49 - 2.58]	<0.001	7.9	3.72 [3.22 - 4.30]	<0.001	15	2.77 [2.24 - 3.43]	<0.001
**95**	17.5	2.03 [1.51 - 2.73]	<0.001	97.21	1.77 [1.29 - 2.44]	0.002	11.4	3.64 [3.11 - 4.25]	<0.001	16	2.40 [1.81 - 3.19]	<0.001

Ascending biomarker and SOFA values were grouped into ventiles (increments of 5%) for all patients, based on respective concentrations or values at baseline. Corresponding cut-offs for each ventile were subsequently identified. 28 day mortality prediction was assessed depending on whether the concentration of each biomarker or score was continuously maintained above respective ventiles from baseline to day 10. Relative risk ratios were calculated for each ventile in order to identify the ratio of the risk in the exposed population (i.e. where biomarkers or scores were continuously maintained above the corresponding ventile) compared to that of the non-exposed population (i.e. where biomarkers or scores were below the respective ventile at one time point or more). The χ^2 test was subsequently performed to determine the significance of any difference between the the two populations.

MR-proADM: Mid-regional proadrenomedullin; *PCT*: Procalcitonin; *SOFA*: Sequential Organ Failure Assessment score

Table S14. **7 day, ICU and Hospital mortality rates following PCT and MR-proADM kinetics between baseline and day 1**

	Biomarker kinetics		7 day mortality			ICU mortality			Hospital mortality		
	Baseline	Day 1	N	%	HR [95% CI]	N	%	HR [95% CI]	N	%	HR [95% CI]
PCT decrease ≥20%			461	6.1%		456	16.7%		439	24.1%	
MR-proADM severity level	Low	Low	126	2.4%	1.9 [0.5 - 6.9]*	126	4.8%	3.9 [1.6 - 9.6]*	123	7.3%	4.9 [2.3 - 10.3]*
	Intermediate	Intermediate	205	4.4%	8.2 [3.4 - 21.2]**	202	16.3%	8.7 [3.7 - 20.7]**	194	27.8%	6.2 [2.5 - 14.9]**
	High	High	27	29.6%	15.2 [4.0 - 57.3]***	27	63.0%	34.0 [11.0 - 105.5]***	27	70.4%	30.1 [10.3 - 87.6]***
	Increasing										
	Low	Intermediate	3	0.0%	-	2	0.0%	-	2	0.0%	-
	Intermediate	High	10	20.0%	4.7 [1.0 - 21.6]††	10	30.0%	2.2 [0.5 - 8.9]††	10	50.0%	2.6 [0.7 - 9.3]††
	Decreasing										
	High	Intermediate	30	16.7%	0.5 [0.2 - 1.6]‡	29	37.9%	0.4 [0.1 - 1.1]‡	28	46.4%	0.4 [0.1 - 1.1]‡
	Intermediate	Low	60	1.7%	0.4 [0.0 - 3.0]‡‡	59	10.2%	0.6 [0.1 - 1.5]‡‡	55	10.9%	0.3 [0.1 - 0.8]‡‡
PCT decrease <20%			526	13.7%		517	30.2%		493	36.9%	
MR-proADM severity level	Low	Low	107	5.6%	2.0 [0.8 - 4.9]*	107	10.3%	3.4 [1.7 - 6.8]*	102	13.7%	3.6 [1.9 - 6.8]*
	Intermediate	Intermediate	230	10.9%	2.6 [1.5 - 4.7]**	225	28.0%	3.0 [1.8 - 5.2]**	216	36.6%	2.4 [1.4 - 4.2]**
	High	High	77	26.0%	5.3 [2.1 - 13.2]***	74	54.1%	10.3 [4.7 - 22.3]***	72	58.3%	8.8 [4.2 - 18.3]***
	Increasing										
	Low	Intermediate	30	13.3%	2.5 [0.7 - 8.9]†	29	31.0%	3.9 [1.4 - 10.7]†	27	37.0%	3.7 [1.4 - 9.7]†
	Intermediate	High	46	28.3%	3.0 [1.5 - 5.8]††	45	57.8%	3.3 [1.7 - 6.4]††	43	65.1%	3.2 [1.6 - 6.4]††
	Decreasing										
	High	Intermediate	11	36.6%	0.5 [0.2 - 1.6]‡	11	54.5%	1.0 [0.3 - 3.7]‡	10	80.0%	-
	High	Low	1	0.0%	-	1	0.0%	-	1	0.0%	-
	Intermediate	Low	24	0.0%	0.1 [0.0 - 0.6]‡‡	24	4.2%	0.1 [0.0 - 0.8]‡‡	22	4.5%	0.1 [0.0 - 0.6]‡‡

Hazard ratios for patients with: * continuously intermediate vs. low values; ** continuously high vs. intermediate values; *** continuously high vs. low values; † increasing low to intermediate vs. continuously low values; †† increasing intermediate to high vs. continuously intermediate values; ‡ decreasing high to intermediate vs. continuously high values; ‡‡ decreasing intermediate to low vs. continuously intermediate values.

CI: Confidence Interval; *HR*: Hazard Ratio; *IQR*: Interquartile range; *MR-proADM*: Mid-regional proadrenomedullin; *N*: Number; *PCT*: Procalcitonin

Table S15. ICU and Hospital mortality rates following PCT and MR-proADM kinetics between baseline and day 4

	Biomarker kinetics		ICU mortality			Hospital mortality		
	Baseline	Day 4	N	%	HR [95% CI]	N	%	HR [95% CI]
PCT decrease ≥50%			555	16.8%		532	24.1%	
MR-proADM severity level	Low	Low	114	2.6%	6.9 [2.1 - 23.1]*	109	2.8%	13.3 [4.1 - 43.8]*
	Intermediate	Intermediate	208	15.9%	8.1 [3.8 - 17.2]**	197	27.4%	5.1 [2.4 - 10.7]**
	High	High	38	60.5%	56.2 [15.0 - 210.2]***	38	65.8%	67.9 [18.0 - 256.6]***
	Low	Intermediate	24	29.2%	15.1 [3.6 - 64.1]†	24	33.3%	17.7 [4.2 - 73.6]†
	Intermediate	High	23	43.5%	4.1 [1.7 - 10.0]††	23	56.5%	3.4 [1.4 - 8.3]††
	High	Intermediate	41	22.0%	0.2 [0.1 - 0.5]‡	39	33.3%	1.3 [0.6 - 2.7]‡
	High	Low	3	0.0%	-	2	50.0%	-
	Intermediate	Low	103	8.7%	0.5 [0.2 - 1.0]‡‡	99	11.1%	0.3 [0.2 - 0.7]‡‡
PCT decrease <50%			204	28.9%		194	30.4%	
MR-proADM severity level	Low	Low	56	1.8%	28.1 [3.7 - 216.3]*	54	7.4%	10.1 [3.3 - 31.2]*
	Intermediate	Intermediate	68	33.8%	1.8 [0.7 - 4.8]**	65	44.6%	1.9 [0.7 - 5.2]**
	High	High	21	47.6%	50.0 [5.8 - 431.5]***	20	60.0%	18.8 [4.8 - 72.7]***
	Low	Intermediate	16	43.7%	42.8 [4.7 - 390.2]†	14	57.1%	16.7 [3.8 - 72.4]†
	Low	High	4	0.0%	-	4	25.0%	-
	Intermediate	High	29	58.6%	2.8 [1.1 - 6.8]††	28	64.3%	2.2 [0.9 - 5.6]††
	High	Intermediate	-	-		-	-	-
	High	Low	-	-		-	-	-
	Intermediate	Low	10	10.0%	-	9	33.3%	-

Hazard ratios for patients with: * continuously intermediate vs. low values; ** continuously high vs. intermediate values; *** continuously high vs. low values; † Increasing low to intermediate vs. continuously low values; †† Increasing intermediate to high vs. continuously intermediate values; ‡ decreasing high to intermediate vs. continuously high values; ‡‡ Decreasing intermediate to low vs. continuously intermediate values.

CI: Confidence Interval; *HR*: Hazard Ratio; *IQR*: Interquartile range; *MR-proADM*: Mid-regional proadrenomedullin; *N*: Number; *PCT*: Procalcitonin

Table S16. Baseline biomarker and clinical score correlation with SOFA at baseline and SOFA at day 1

Baseline biomarkers and scores	Baseline SOFA			Day 1 SOFA		
	Patients (N)	Correlation [95% CI]	p-value	Patients (N)	Correlation [95% CI]	p-value
**MR-proADM**	969	0.47 [0.41 - 0.51]	<0.001	969	0.57 [0.52 - 0.61]	<0.001
**PCT**	1007	0.23 [0.17 - 0.29]	<0.001	969	0.22 [0.16 - 0.28]	<0.001
**CRP**	918	0.06 [0.00 - 0.13]	0.006	885	0.04 [0.00 - 0.12]	0.271
**Lactate**	1044	0.33 [0.27 - 0.38]	<0.001	1005	0.40 [0.35 - 0.45]	<0.001
**SAPS II**	1051	0.60 [0.56 - 0.64]	<0.001	1011	0.50 [0.45 - 0.54]	<0.001
**APACHE II**	1051	0.62 [0.58 - 0.65]	<0.001	1011	0.53 [0.48 - 0.57]	<0.001

APACHE II: Acute Physiological and Chronic Health Evaluation II score; *CI*: Confidence Interval; *CRP*: C-reactive protein; *HR*: Hazard Ratio; *IQR*: Interquartile range; *MR-proADM*: Mid-regional proadrenomedullin; *N*: Number; *PCT*: Procalcitonin; *SAPS II*: Simplified Acute Physiological II score; *SOFA*: Sequential Organ Failure Assessment score

Table S17. **Baseline MR-proADM correlations with SOFA subscores at baseline and on day 1**

	Baseline SOFA			Day 1 SOFA		
SOFA subscore	Patients (N)	Correlation [95% CI]	p-value	Patients (N)	Correlation [95% CI]	p-value
**Circulation**	1022	0.18 [0.12 - 0.23]	<0.001	995	0.23 [0.17 - 0.29]	<0.001
**Pulmonary**	1025	0.12 [0.06 - 0.18]	<0.001	994	0.15 [0.09 - 0.21]	<0.001
**Coagulation**	1028	0.30 [0.25 - 0.36]	<0.001	1002	0.40 [0.35 - 0.45]	<0.001
**Renal**	1030	0.50 [0.45 - 0.54]	<0.001	1001	0.62 [0.58 - 0.66]	<0.001
**Liver**	1014	0.20 [0.14 - 0.26]	<0.001	993	0.36 [0.30 - 0.40]	<0.001
**CNS**	1030	0.03 [-0.03 - 0.09]	0.386	1003	0.08 [0.02 - 0.14]	0.009

CI: Confidence Interval; *N*: Number; *SOFA*: Sequential Organ Failure Assessment score

Table S18. **Biomarker correlations with SOFA scores throughout ICU treatment**

		MR-proADM	PCT	CRP	Lactate
**Day 1**	Patients (N)	960	960	894	1008
	Correlation [95% CI]	0.51 [0.46 - 0.55]	0.24 [0.18 - 0.30]	-0.04 [-0.10 - 0.03]	0.48 [0.43 - 0.53]
	p-value	<0.001	<0.001	<0.001	<0.001
**Day 4**	Patients (N)	729	729	667	754
	Correlation [95% CI]	0.58 [0.53 - 0.63]	0.13 [0.06 - 0.20]	0.14 [0.06 - 0.21]	0.36 [0.29 - 0.42]
	p-value	<0.001	<0.001	<0.001	<0.001
**Day 7**	Patients (N)	580	581	547	612
	Correlation [95% CI]	0.58 [0.53 - 0.64]	0.05 [-0.03 - 0.13]	0.15 [0.07 - 0.23]	0.43 [0.37 - 0.50]
	p-value	<0.001	0.237	<0.001	<0.001
**Day 10**	Patients (N)	473	473	429	483
	Correlation [95% CI]	0.65 [0.59 - 0.70]	0.28 [0.20 - 0.37]	0.13 [0.03 - 0.22]	0.34 [0.26 - 0.42]
	p-value	<0.001	<0.001	0.008	<0.001

CI: Confidence Interval; *CRP*: C-reactive protein; *N*: Number; *MR-proADM*: Mid-regional proadrenomedullin; *PCT*: Procalcitonin; *SOFA*: Sequential Organ Failure Assessment score

Table S19. **Time dependent Cox regressions for single and cumulative additions of MR-proADM**

	Bivariate model					Multivariate model				
Addition of subsequent single day measurements to baseline values	LR χ^2	DF	Added LR χ^2	Added DF	p-value	LR χ^2	DF	Added LR χ^2	Added DF	p-value
MR-proADM baseline	144.2	1	Reference			163.0	10	Reference		
+ Day 1 measurement	169.8	2	25.6	1	<0.001	190.6	11	27.6	1	<0.001
+ Day 4 measurement	161.9	2	17.7	1	<0.001	180.4	11	17.4	1	<0.001
+ Day 7 measurement	175.7	2	31.5	1	<0.001	195.1	11	32.1	1	<0.001
+ Day 10 measurement	179.8	2	35.6	1	<0.001	197.9	11	34.9	1	<0.001
Addition of subsequent cumulative day measurements to baseline values										
MR-proADM baseline	144.2	1	Reference			163.0	10	Reference		
+ Day 1	169.8	2	25.6	1	<0.001	190.6	11	27.6	1	<0.001
+ Day 1 + Day 4	174.9	3	5.1	1	0.024	195.4	12	4.8	1	0.028
+ Day 1 + Day 4 + Day 7	188.7	4	13.9	1	<0.001	210.4	13	15.0	1	<0.001
+ Day 1 + Day 4 + Day 7 + Day 10	195.2	5	6.5	1	0.011	216.6	14	6.2	1	0.013

DF: Degrees of Freedom; *MR-proADM*: Mid-regional proadrenomedullin

3. Supplementary Figures

Figure S1. 28 and 90 day mortality rates following PCT and MR-proADM kinetics between baseline and day 4

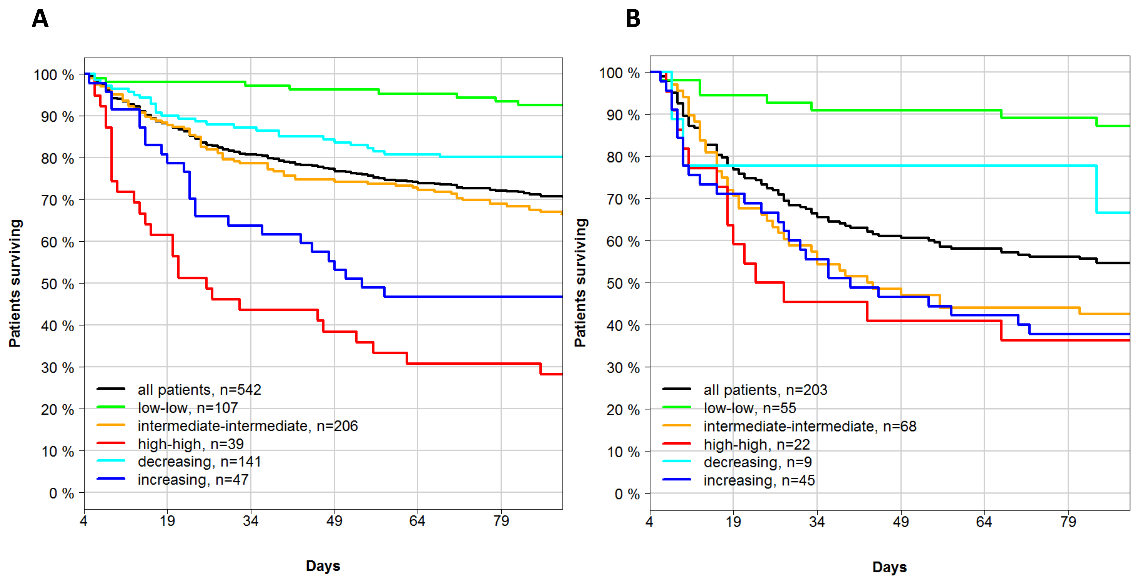

	Biomarker Kinetics		28 day mortality			90 day mortality		
	Baseline	Day 4	N	%	HR [95% CI]	N	%	HR [95% CI]
PCT decrease ≥50%			557	17.1%		542	29.3%	
MR-proADM severity level	Low	Low	111	1.8%	11.2 [2.7 - 46.4]*	107	7.5%	5.3 [2.5 - 10.9]*
	Intermediate	Intermediate	209	18.7%	3.8 [2.3 - 6.5]**	206	33.5%	3.3 [2.1 - 5.1]**
	High	High	39	53.8%	43.1 [10.1 - 184.0]***	39	71.8%	17.4 [7.9 - 38.2]***
	Increasing							
	Low	Intermediate	24	25.0%	15.6 [3.1 - 77.2]†	24	41.7%	7.1 [2.8 - 17.9]†
	Intermediate	High	23	43.5%	2.6 [1.3 - 5.3]††	23	65.2%	2.6 [1.5 - 4.5]††
	Decreasing							
	High	Intermediate	42	21.4%	0.3 [0.1 - 0.7]‡	41	36.6%	0.3 [0.2 - 0.6]‡
	High	Low	3	0.0%	-	2	50.0%	-
	Intermediate	Low	105	7.6%	0.4 [0.2 - 0.8]‡‡	100	13.0%	0.3 [0.2 - 0.6]‡‡
PCT decrease <50%			210	29.5%		203	45.5%	
MR-proADM severity level	Low	Low	56	7.1%	6.3 [2.2 - 18.1]*	55	12.7%	6.2 [2.8 - 13.9]*
	Intermediate	Intermediate	70	38.6%	1.5 [0.8 - 3.0]**	68	57.4%	1.3 [0.7 - 2.3]**
	High	High	23	52.2%	9.5 [3.1 - 29.5]***	22	63.6%	7.9 [3.2 - 19.5]***
	Increasing							
	Low	Intermediate	17	17.6%	2.8 [0.6 - 12.5]†	15	53.3%	5.5 [2.0 - 15.2]†
	Low	High	4	0.0%	-	4	25.0%	-
	Intermediate	High	30	46.7%	1.4 [0.7 - 2.6]††	30	66.7%	1.3 [0.8 - 2.2]††
	Decreasing							
	Intermediate	Low	10	20.0%	-	9	33.4%	-

Kaplan-Meier plots illustrate patient subgroups stratified by MR-proADM severity levels for 90 day mortality, based on corresponding PCT concentrations from baseline to day 4, either decreasing by (A) ≥50%, or (B) <50%. Severity levels are grouped either as continuously low, intermediate or high, or as a composite for increasing or decreasing levels. Individual Hazard ratios for comparisons between patient subgroups are indicated by: * continuously intermediate vs. low values; ** continuously high vs. intermediate values; *** continuously high vs. low values; † Increasing low to intermediate vs. continuously low values; †† Increasing intermediate to high vs. continuously intermediate values; ‡ decreasing high to intermediate vs. continuously high values; ‡‡ Decreasing intermediate to low vs. continuously intermediate values.

CI: Confidence Interval; *HR*: Hazard Ratio; *IQR*: Interquartile range; *MR-proADM*: Mid-regional proadrenomedullin; *N*: Number; *PCT*: Procalcitonin

Figure S2. **Influence of infectious origin on 28 day mortality prediction**

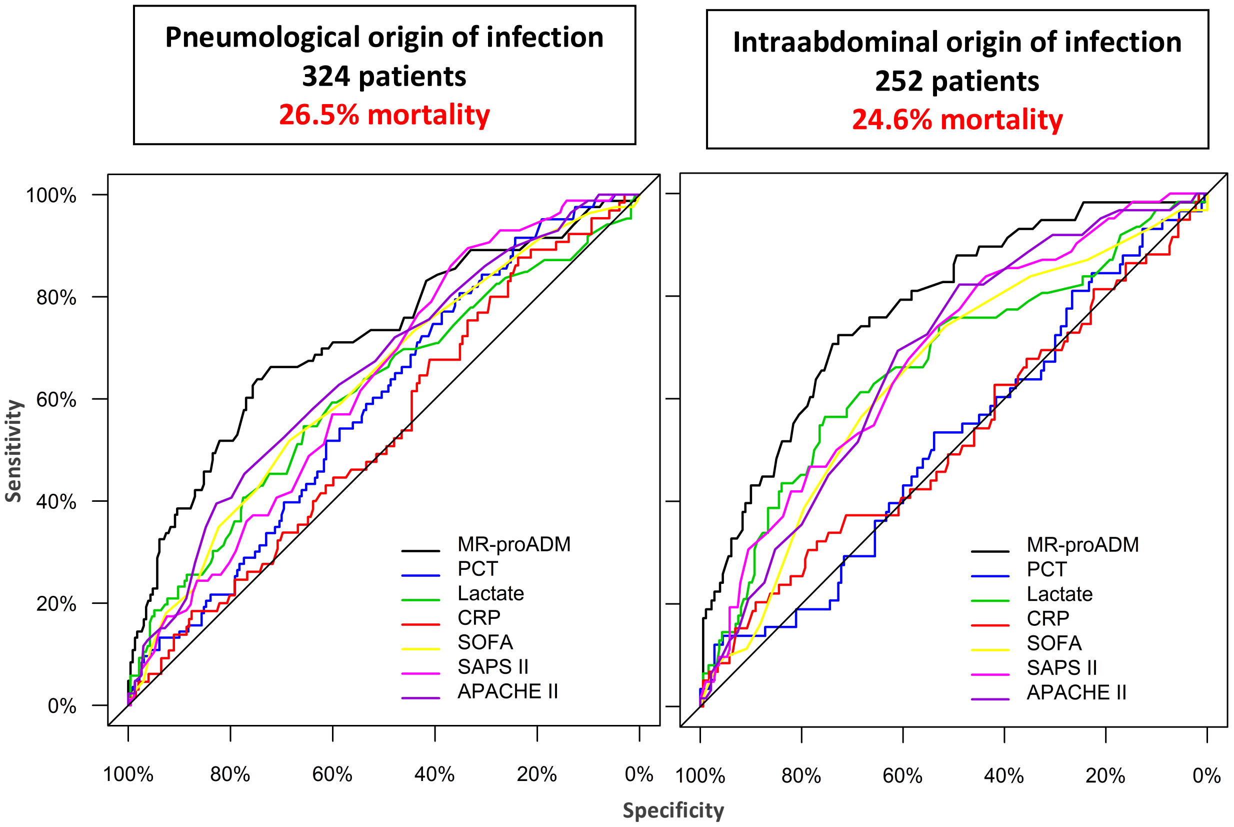

					Univariate Cox regression				Multivariate Cox regression			
	Biomarker or clinical score	Patients (N)	Mortality (N)	AUROC	LR χ^2	C-index	HR IQR [95% CI]	p-value	LR χ^2	C-index	HR IQR [95% CI]	p-value
Pneumological	MR-proADM	313	83	0.72	37.9	0.69	2.7 [2.0 - 3.7]	<0.001	45.1	0.71	2.5 [1.7 - 3.6]	<0.001
	PCT	313	83	0.59	6.4	0.58	1.6 [1.1 - 2.2]	0.011	26.0	0.66	1.5 [1.1 - 2.2]	0.004
	CRP	267	65	0.46	0.8	0.53	0.9 [0.7 - 1.1]	0.375	14.7	0.63	0.9 [0.7 - 1.1]	0.142
	Lactate	322	86	0.61	12.6	0.61	1.6 [1.2 - 2.1]	<0.001	30.1	0.67	1.5 [1.1 - 2.0]	0.001
	SOFA	315	83	0.63	12.4	0.62	1.7 [1.3 - 2.3]	<0.001	29.6	0.68	1.6 [1.1 - 2.2]	0.001
	SAPS II	324	86	0.63	13.2	0.62	1.6 [1.3 - 2.1]	<0.001	28.8	0.67	1.5 [1.1 - 1.9]	0.001
	APACHE II	324	86	0.63	19.5	0.64	1.9 [1.4 - 2.5]	<0.001	33.4	0.68	1.7 [1.3 - 2.3]	<0.001
Intraabdominal	MR-proADM	238	58	0.78	47.4	0.75	4.5 [2.9 - 7.1]	<0.001	55.7	0.76	4.8 [2.9 - 8.0]	<0.001
	PCT	238	58	0.52	0.4	0.52	1.1 [0.8 - 1.7]	0.525	15.0	0.64	1.2 [0.8 - 1.9]	0.131
	CRP	233	59	0.48	0.1	0.53	1.0 [0.8 - 1.3]	0.781	12.0	0.62	1.1 [0.8 - 1.4]	0.286
	Lactate	249	62	0.67	18.0	0.66	2.2 [1.5 - 3.0]	<0.001	28.2	0.70	2.1 [1.5 - 3.0]	0.002
	SOFA	248	62	0.66	8.9	0.63	1.5 [1.2 - 2.0]	0.003	18.3	0.64	1.5 [1.1 - 2.0]	0.049
	SAPS II	252	62	0.68	17.9	0.66	1.9 [1.4 - 2.6]	<0.001	24.3	0.67	1.9 [1.3 - 2.6]	0.007
	APACHE II	252	62	0.68	14.6	0.65	1.8 [1.3 - 2.3]	<0.001	20.6	0.66	1.6 [1.2 - 2.2]	0.024

Patients with mixed origins of infection were excluded from the analysis.

APACHE II: Acute Physiological and Chronic Health Evaluation II score; *CI*: Confidence Interval; *CRP*: C-reactive protein; *HR*: Hazard Ratio; *IQR*: Interquartile range; *MR-proADM*: Mid-regional proadrenomedullin; *N*: Number; *PCT*: Procalcitonin; *SAPS II*: Simplified Acute Physiological II score; *SOFA*: Sequential Organ Failure Assessment score

Figure S3. **Influence of microbial species on 28 day mortality prediction**

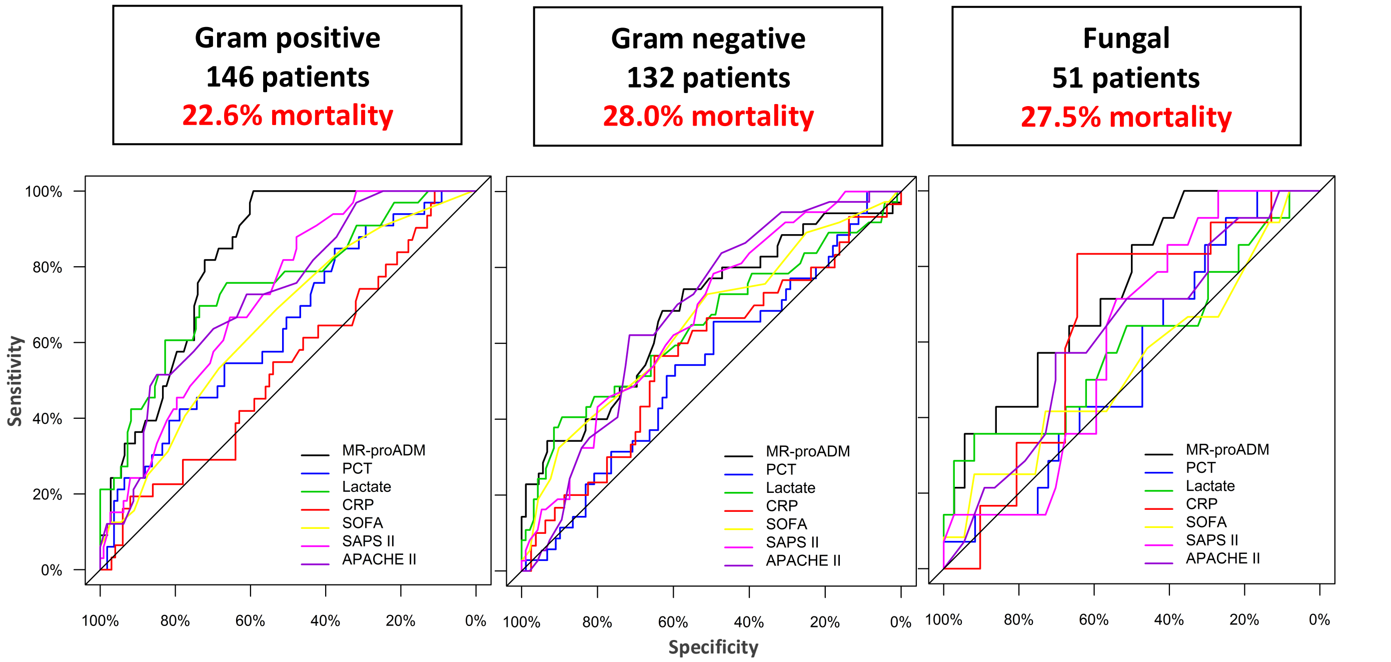

	Biomarker or clinical score	Patients (N)	Mortality (N)	AUROC	Univariate Cox regression LR χ^2	C-index	HR IQR [95% CI]	p-value	Multivariate Cox regression LR χ^2	C-index	HR IQR [95% CI]	p-value
Gram positive	**MR-proADM**	141	33	0.82	37.2	0.81	5.0 [2.9 - 8.6]	<0.001	50.0	0.84	5.0 [2.7 - 9.2]	<0.001
	**PCT**	142	33	0.64	7.9	0.64	2.4 [1.3 - 4.4]	0.005	30.3	0.76	3.0 [1.5 - 5.7]	<0.001
	**CRP**	131	31	0.54	0.2	0.51	0.9 [0.7 - 1.3]	0.656	19.8	0.71	1.0 [0.7 - 1.4]	0.031
	**Lactate**	143	33	0.75	28.9	0.74	4.6 [2.6 - 8.1]	<0.001	44.9	0.83	5.0 [2.6 - 9.7]	<0.001
	**SOFA**	143	32	0.66	8.8	0.65	1.9 [1.3 - 2.8]	0.003	31.8	0.76	2.7 [1.6 - 4.6]	<0.001
	**SAPS II**	146	33	0.72	16.8	0.71	2.9 [1.7 - 4.7]	<0.001	28.4	0.76	2.7 [1.5 - 4.9]	0.002
	**APACHE II**	146	33	0.73	17.3	0.71	2.4 [1.6 - 3.5]	<0.001	33.1	0.77	2.8 [1.7 - 4.7]	<0.001
Gram negative	**MR-proADM**	124	35	0.69	12.1	0.68	2.3 [1.4 - 3.8]	<0.001	26.0	0.75	2.2 [1.2 - 3.8]	0.004
	**PCT**	124	35	0.54	0.6	0.54	1.2 [0.7 - 2.1]	0.458	17.8	0.67	1.2 [0.7 - 2.3]	0.058
	**CRP**	110	30	0.57	0.4	0.56	1.2 [0.7 - 1.8]	0.526	17.1	0.68	1.4 [0.9 - 2.2]	0.073
	**Lactate**	131	37	0.65	10.0	0.64	1.9 [1.3 - 2.8]	0.002	23.4	0.71	1.7 [1.1 - 2.7]	0.009
	**SOFA**	129	37	0.65	9.0	0.64	1.8 [1.2 - 2.7]	0.003	25.5	0.72	1.9 [1.2 - 2.9]	0.005
	**SAPS II**	132	37	0.67	9.9	0.65	1.9 [1.3 - 2.8]	0.002	25.1	0.71	1.9 [1.2 - 3.0]	0.005
	**APACHE II**	132	37	0.69	7.9	0.66	1.7 [1.2 - 2.4]	0.005	22.3	0.70	1.7 [1.1 - 2.6]	0.014
Fungal	**MR-proADM**	50	14	0.74	7.9	0.69	2.5 [1.3 - 4.9]	0.005	14.4	0.78	3.4 [1.1 - 10.7]	0.155
	**PCT**	50	14	0.46	0.3	0.52	1.3 [0.5 - 3.0]	0.610	8.5	0.72	1.1 [0.4 - 3.0]	0.579
	**CRP**	43	12	0.65	0.6	0.65	0.8 [0.5 - 1.3]	0.440	14.7	0.81	0.5 [0.2 - 1.2]	0.143
	**Lactate**	51	14	0.60	2.7	0.59	2.0 [0.9 - 4.7]	0.103	13.2	0.74	3.3 [1.0 - 11.0]	0.213
	**SOFA**	49	12	0.54	0.8	0.54	1.4 [0.7 - 2.8]	0.367	7.1	0.73	1.1 [0.5 - 2.8]	0.716
	**SAPS II**	51	14	0.60	2.2	0.60	1.5 [0.9 - 2.6]	0.141	10.0	0.75	1.4 [0.7 - 2.8]	0.443
	**APACHE II**	51	14	0.62	1.6	0.62	1.6 [0.8 - 3.3]	0.205	10.1	0.76	1.7 [0.7 - 4.4]	0.432

Patients with mixed microbial infections were excluded from the analysis.

APACHE II: Acute Physiological and Chronic Health Evaluation II score; *CI*: Confidence Interval; *CRP*: C-reactive protein; *HR*: Hazard Ratio; *IQR*: Interquartile range; *MR-proADM*: Mid-regional proadrenomedullin; *N*: Number; *PCT*: Procalcitonin; *SAPS II*: Simplified Acute Physiological II score; *SOFA*: Sequential Organ Failure Assessment score

Figure S4. **Influence of mode of ICU entry on 28 day mortality prediction**

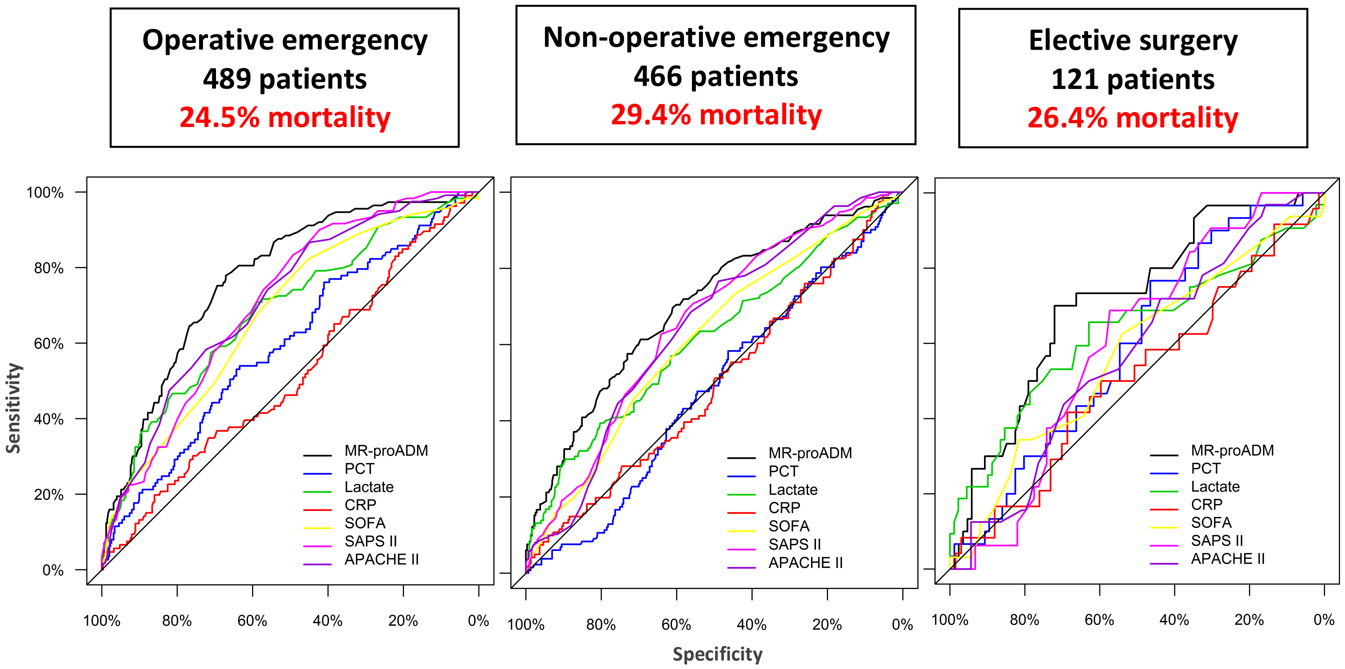

	Biomarker or clinical score	Patients (N)	Mortality (N)	AUROC	Univariate Cox regression				Multivariate Cox regression			
					LR χ^2	C-index	HR IQR [95% CI]	p-value	LR χ^2	C-index	HR IQR [95% CI]	p-value
**Operative**	**MR-proADM**	466	113	0.77	87.4	0.75	4.1 [3.0 - 5.6]	<0.001	106.4	0.77	3.8 [2.8 - 5.3]	<0.001
	**PCT**	466	113	0.60	11.8	0.59	1.6 [1.2 - 2.2]	<0.001	53.1	0.70	1.7 [1.3 - 2.4]	<0.001
	**CRP**	421	106	0.48	1.2	0.52	1.1 [0.9 - 1.4]	0.270	39.7	0.68	1.2 [0.9 - 1.4]	<0.001
	**Lactate**	483	120	0.68	46.4	0.67	2.4 [1.9 - 3.1]	<0.001	73.7	0.71	2.3 [1.8 - 3.0]	<0.001
	**SOFA**	482	118	0.68	34.9	0.65	2.0 [1.6 - 2.4]	<0.001	65.7	0.71	2.0 [1.6 - 2.5]	<0.001
	**SAPS II**	489	120	0.71	50.5	0.68	2.2 [1.8 - 2.7]	<0.001	65.9	0.70	2.0 [1.6 - 2.5]	<0.001
	**APACHE II**	489	120	0.71	47.8	0.68	2.3 [1.8 - 2.8]	<0.001	64.8	0.71	2.0 [1.6 - 2.5]	<0.001
**Non-operative**	**MR-proADM**	448	132	0.70	48.6	0.68	2.6 [2.0 - 3.4]	<0.001	56.5	0.69	2.4 [1.8 - 3.3]	<0.001
	**PCT**	449	132	0.52	0.8	0.52	1.1 [0.9 - 1.5]	0.364	24.4	0.62	1.1 [0.8 - 1.4]	0.007
	**CRP**	424	121	0.50	0.2	0.49	1.0 [0.8 - 1.2]	0.628	23.6	0.62	1.0 [0.8 - 1.2]	0.009
	**Lactate**	462	137	0.62	24.5	0.62	1.9 [1.5 - 2.4]	<0.001	43.7	0.67	1.8 [1.4 - 2.3]	<0.001
	**SOFA**	450	132	0.62	15.9	0.61	1.7 [1.3 - 2.1]	<0.001	39.5	0.66	1.7 [1.3 - 2.2]	<0.001
	**SAPS II**	466	137	0.65	25.4	0.64	1.6 [1.3 - 1.9]	<0.001	43.4	0.66	1.5 [1.3 - 1.8]	<0.001
	**APACHE II**	466	137	0.64	23.9	0.63	1.7 [1.4 - 2.1]	<0.001	40.2	0.66	1.6 [1.3 - 2.0]	<0.001
**Elective**	**MR-proADM**	116	30	0.71	12.1	0.69	2.8 [1.6 - 5.2]	<0.001	17.3	0.72	2.3 [1.2 - 4.5]	0.044
	**PCT**	116	30	0.59	3.3	0.59	1.6 [1.0 - 2.6]	0.068	15.1	0.70	1.7 [1.0 - 2.8]	0.087
	**CRP**	91	24	0.51	0.0	0.50	1.0 [0.7 - 1.4]	0.865	11.5	0.70	0.8 [0.5 - 1.3]	0.322
	**Lactate**	121	32	0.63	9.5	0.63	2.2 [1.4 - 3.6]	0.002	21.0	0.72	2.2 [1.3 - 3.6]	0.021
	**SOFA**	119	32	0.58	0.9	0.56	1.2 [0.9 - 1.6]	0.348	13.7	0.69	1.0 [0.7 - 1.3]	0.186
	**SAPS II**	121	32	0.60	1.4	0.59	1.3 [0.9 - 1.9]	0.233	13.1	0.68	0.9 [0.6 - 1.5]	0.218
	**APACHE II**	121	32	0.57	1.1	0.57	1.3 [0.8 - 1.9]	0.295	13.1	0.69	0.9 [0.6 - 1.5]	0.216

APACHE II: Acute Physiological and Chronic Health Evaluation II score; *CI*: Confidence Interval; *CRP*: C-reactive protein; *HR*: Hazard Ratio; *IQR*: Interquartile range; *MR-proADM*: Mid-regional proadrenomedullin; *N*: Number; *PCT*: Procalcitonin; *SAPS II*: Simplified Acute Physiological II score; *SOFA*: Sequential Organ Failure Assessment score
